# Metabolic alterations in urine extracellular vesicles are associated to prostate cancer pathogenesis and progression

**DOI:** 10.1080/20013078.2018.1470442

**Published:** 2018-05-07

**Authors:** Marc Clos-Garcia, Ana Loizaga-Iriarte, Patricia Zuñiga-Garcia, Pilar Sánchez-Mosquera, Ana Rosa Cortazar, Esperanza González, Verónica Torrano, Cristina Alonso, Miriam Pérez-Cormenzana, Aitziber Ugalde-Olano, Isabel Lacasa-Viscasillas, Azucena Castro, Felix Royo, Miguel Unda, Arkaitz Carracedo, Juan M. Falcón-Pérez

**Affiliations:** a CIC bioGUNE, Bizkaia Technology Park, Derio, Spain; b Department of Urology, Basurto University Hospital, Bilbao, Spain; c Centro de Investigación Biomédica en Red de Cáncer (CIBERONC); d OWL Metabolomics, Bizkaia Technology Park, Derio, Spain; e Department of Pathology, Basurto University Hospital, Bilbao, Spain; f Centro de Investigación Biomédica en Red de Enfermedades Hepáticas y Digestivas (CIBEREHD); g Ikerbasque, Basque foundation for science, Bilbao, Spain; h Biochemistry and Molecular Biology Department, University of the Basque Country (UPV/EHU), Bilbao, Spain

**Keywords:** Prostate, urine, exosomes, metabolomics, metabolism, biomarkers

## Abstract

Urine contains extracellular vesicles (EVs) that concentrate molecules and protect them from degradation. Thus, isolation and characterisation of urinary EVs could increase the efficiency of biomarker discovery. We have previously identified proteins and RNAs with differential abundance in urinary EVs from prostate cancer (PCa) patients compared to benign prostate hyperplasia (BPH). Here, we focused on the analysis of the metabolites contained in urinary EVs collected from patients with PCa and BPH. Targeted metabolomics analysis of EVs was performed by ultra-high-performance liquid chromatography–mass spectrometry. The correlation between metabolites and clinical parameters was studied, and metabolites with differential abundance in PCa urinary EVs were detected and mapped into cellular pathways. We detected 248 metabolites belonging to different chemical families including amino acids and various lipid species. Among these metabolites, 76 exhibited significant differential abundance between PCa and BPH. Interestingly, urine EVs recapitulated many of the metabolic alterations reported in PCa, including phosphathidylcholines, acyl carnitines, citrate and kynurenine. Importantly, we found elevated levels of the steroid hormone, 3beta-hydroxyandros-5-en-17-one-3-sulphate (dehydroepiandrosterone sulphate) in PCa urinary EVs, in line with the potential elevation of androgen synthesis in this type of cancer. This work supports urinary EVs as a non-invasive source to infer metabolic changes in PCa.

## Introduction

Prostate cancer (PCa) is among the most frequently diagnosed and deadly types of cancer in men in Western countries (http://globocan.iarc.fr). Lack of sensitive and specific diagnostic tools, especially to detect early stages of the disease, and the unknown underlying mechanisms of onset and progression of PCa are the major problems to treat PCa with the highest efficacy. Thus, there is a high demand to discover more sensitive and specific biomarkers to improve PCa diagnosis and prognosis. Nowadays, prostate-specific antigen (PSA) blood screening tests, together with clinical T-stage and Gleason score are the standard tests to discriminate patients with low, intermediate or high risk to suffer PCa [1].

Metabolomics is recognised as the ultimate “omics” discipline with high potential to identify sensitive and specific markers, and to understand the mechanisms involved in the development of pathological processes [2]. The recent technological revolution in separation and detection of small molecules, combined with rapid progress in bioinformatics, is making possible to rapidly measure a large number of metabolites in a small amount of sample [3,4]. Metabolomics comprises the qualitative and quantitative measurement of the metabolic response to physiological or pathological stimuli. It involves the extraction and measurement of low molecular weight molecules (e.g. amino acids, sugars, bile acids, fatty acids, vitamins, etc.) belonging to different metabolic pathways to generate metabolic profiles of cells, tissues or biofluids [5,6]. Previous studies have shown the utility of serum metabolite levels as a diagnostic tool for different cancer types [7], and in PCa some metabolites have already been suggested as candidate biomarkers. Increased serum levels of polyunsaturated fatty acids have been associated to reduce risk of PCa, while higher levels of serum testosterone were associated with an increased risk of suffering this malignancy [8]. Other metabolomics approaches have reported alterations of acyl carnitines, glucose, glycerophospholipids (including lysophosphatidylcholines and phosphatidylcholines), amino acids and triglycerides in PCa [9].

Urine samples have been intensely used to identify PCa biomarkers [10], due to its easy availability and handling, and its anatomical proximity to the prostate. As occurs for the serum, there are also several metabolomics studies of urine samples that found alterations in urinary levels of more than 20 metabolites including N-methyl glycine, kynurenine, uracil, glycerol 3-phosphate, dihydroxybutanoic acid, xylonic acid, pyrimidine, ribofuranoside and xylopyranose (reviewed in [11]). These studies have pointed out that many metabolic pathways may be altered in PCa including glycine synthesis and degradation, and carbohydrate and energy metabolisms. Although all these metabolites need further clinical validation, they support the notion that metabolomics constitutes a suitable technology to identify candidate biomarkers of PCa.

One important drawback of using urine sample for biomarker discovery is that many of their constituents are diluted avoiding to be detected by current technologies. Thus, in order to detect underrepresented molecules, it is still required to concentrate the sample. In this context, cell-secreted extracellular vesicles (EVs) are present in all body fluids, including urine [12], and could provide a concentrated source of molecules. Thus, a deep analysis of the urinary EVs composition could open a window of opportunities to identify more sensitive and specific PCa biomarkers. In line, a recent lipidomics study performed in these urinary vesicles from healthy and PCa samples reveal up to nine lipid species differentially expressed as potential PCa biomarkers [13] supporting the existence of metabolic changes in urine EVs from PCa patients.

In the current study, we have compared urinary EVs obtained from PCa and benign prostate hyperplasia (BPH) patients, and focused on the analysis of the metabolites that they contain by performing an UHPLC-MS targeted metabolomics analysis. We evaluated the levels of 248 metabolites belonging to different chemical nature including amino acids, nucleosides, vitamins, as well as different lipid species. Among them, 76 metabolites were found significantly altered in PCa compared to BPH. Some of these metabolites were significantly correlated with current markers of PCa (e.g. PSA). Interestingly, dehydroepiandrosterone sulphate was among the most significantly altered metabolites in PCa, supporting the notion that beyond their function as “metabolic machines” [4,14,15], EVs could inform about metabolic alterations of cancerous tissue.

## Materials and methods

### Patient samples

All urine samples were obtained from the Basque Biobank for research (BIOEF, Basurto University hospital) upon informed consent and with evaluation and approval from the corresponding ethics committee (CEIC code OHEUN11-12 and OHEUN14-14). Clinical classification of the patients is described in Table 1. For each sample, urine (50 ml) was collected by spontaneous micturition, centrifuged at 2,000 × g 10 min, filtered through a 0.22 μm-pore membrane and immediately frozen at −80ºC.

**Table 1.** Clinical classification of the samples.

Disease status	Stage	Perineural invasion	*n*
Prostate cancer (PCA) (64 ± 4.41)	Stage 2 (64 ± 4.12)	No (Pn0) (65.5 ± 5.02)	6
		Yes (Pn1) (64 ± 3.47)	10
	Stage 3 (64.5 ± 4.68)	*NA*	15
Benign hyperplasia (BPH) (70 ± 5.71)	*NA*	*NA*	14

In parentheses are indicated the median ± SD of age for each group of samples.

### Urine extracellular vesicle isolation and characterisation

To isolate EVs from urine (average ± SEM; 49.7 ± 0.86 ml), the stored samples were thawed, centrifuged at 10,000 × g for 30 min and the supernatant ultra-centrifuged at 100,000 × g for 75 min. The resulting pellet was washed with an excess of phosphate-saline buffer (PBS), and again ultra-centrifuged at 100,000 × g for 60 min. Final pellet was re-suspended in 150 µL of PBS, aliquot generated and kept at −80°C for further analysis. Protein was determined by Bradford and obtained 32.7 ± 4.6 (mean±SEM) micrograms on average of total purified protein from the initial urine volume (50 ml). Size distribution of the particles present in the isolated preparations was determined by measuring the Brownian motion using a NanoSight LM10 system equipped with a fast video capture and particle-tracking software (Malvern, UK). Pre- and post-acquisition settings were maintained the same for all the samples and each video was analysed to give the mean, mode, and median vesicle size, as well as an estimate of the particle concentration. Then, an average curve was calculated for each group of patients to be compared among them. Cryo-electron microscopy and Western-blot analysis were performed as describe previously [16].

## Metabolite extraction and UHPLC-MS analysis

Metabolic profiles of urinary EVs were semi-quantified using four UHPLC-MS based analytical platforms as previously described [17,18]. Methanol was first added to urinary EV preparations, and after brief vortex, chloroform was added. Both extraction solvents were spiked with metabolites not detected in unspiked EV extracts: tryptophan-d5(indole-d5), PC(13:0/0:0), FA(19:0), dehydrocholic acid, SM(d18:1/6:0), PE(17:0/17:0), PC(19:0/19:0), TAG(13:0/13:0/13:0), Cer(d18:1/17:0), ChoE(12:0), anthranilic acid-(ring-13C6), phenylthiohydantoin (PTH)-valine and glycocholic-2,2,4,4-d4 acid. Samples were incubated at −20°C for 30 min and, after vortex, three different phases were collected. Platform 1 included fatty acyls, bile acids, steroids and lysoglycerophospholipids profiling. Supernatants were collected after centrifugation at 16,000 × g for 15 min, dried, reconstituted in methanol, resuspended for 20 min and centrifuged (16,000 × g for 5 min) before being transferred to vials for UHPLC-MS analysis. Platform 2 included glycerolipids, cholesteryl esters, sphingolipids and glycerophospholipids profiling. Extracts were mixed with water (pH = 9) and after brief vortex mixing, the samples were incubated for 60 min at −20°C. After centrifugation at 16,000 × g for 15 min, the organic phase was collected and the solvent removed. The dried extracts were then reconstituted in acetronitrile/isopropanol (50:50), resuspended for 10 min, centrifuged (16,000 × g for 5 min) and transferred to vials for UHPLC-MS analysis. Platform 3 included amino acids profiling; 10 μl aliquots from the extracts prepared for Platform 1 were transferred to microtubes and derivatised for amino acid analysis. Finally, Platform 4 consisted in the analysis of polar metabolites profiling, including central carbon metabolism. Extracts were mixed with chloroform. After brief vortex mixing, water was added and samples were mixed for 10 min at room temperature. Afterwards, samples were centrifuged at 16,000 × g for 10 min. The supernatants were collected and dried. Extracts were then solubilised in water and after centrifugation, supernatants were transferred to vials for UHPLC-MS analysis.

Chromatographic separation and mass spectrometric detection conditions employed were previously described [17,18]. The overall quality of the analysis procedure was monitored using six repeat injections of a pooled sample, considered as the quality control sample. For each of the four analytical platforms, randomised sample injections were performed, with QC calibration and validation extracts uniformly interspersed throughout the entire batch run. Generally, the retention time stability was <6 s injection-to-injection variation and the mass accuracy <3 ppm for m/z 400–1200, and <1.2 mDa for m/z 50–400. Details of lipid nomenclature used in this work is provided as supplementary material.

## Data processing, statistical and bioinformatics analyses

### Amount of urine sample and data normalisation

A similar volume of urine sample (50 ml) from each patient was employed for obtaining the EV preparations. Then, the complete EV preparations were analysed by UPLC-MS metabolomics analysis. The peak intensities for each metabolite included in the analysis were normalised to the sum of the peak intensities within each sample. There was no significant correlation (F < Fcrit) between the sum of the peak intensities used for the normalisation and the groups being compared in the study.

### Missing values imputation

First, metabolites that were not detected in at least 70% of the whole set of samples were removed from the analysis. Then, taking the minimal value for each metabolite and dividing it by a factor of 10, missing values were imputed in order to obtain the final data set.

### Univariate analysis

Three different comparisons were established for the analyses:

- Prostate cancer (PCa) *vs* benign prostate hyperplasia (BPH).
- PCa pathological stage 3 *vs* PCa pathological stage 2.
- In the PCa pathological stage 2 group, perineural invasion: Pn1 *vs* Pn0.

The mean and 90% Winsorized-mean for each metabolite and each group of patients were calculated, as well as, Student’s *t*-test or Wilcoxon signed-rank test, depending on the normality of the data that was assessed using Shapiro-Wilk test. Median, standard error of the mean (SEM), the standard deviation (SD), coefficient of variation and the Interquartile Range (IQR) were also calculated.

Several calculations were performed for the three distinct comparisons. We calculated the F-test of the two variances, the Student’s *t*-test, Wilcoxon signed-rank and Fold Change for each metabolite. To test the discriminatory capacity of each metabolite for each one of the three comparisons we performed Receiver Operating Characteristic (ROC) analysis, including in the calculations the values of the Area Under the Curve (AUC), sensitivity, specificity, positive predictive value, negative predictive value, Youden index and the optimal cut-off.

For each one of the three pairwise comparisons, we generated box-plots for those metabolites with significant differences between the two groups with adjusted *p*-values following Bonferroni methodology. Heatmaps indicating log_2_ value of Fold Change and Bonferroni adjusted *p*-values were also calculated. Finally, volcano plots were generated with the log_2_ Fold Change values and Bonferroni adjusted *p*-values.

All statistical analyses were performed using R software v3.3.2 (R Development Core Team, 2016; http://cran.r-project.org) with stats, caret, psych and OptimalCutpoints package [19]. Boxplots and volcano plots were generated with ggplot2 R package. Correlations with clinical parameters such as BMI were done with cor.test function in R software, using Spearman’s method. Both *rho* and *p*-value for each metabolite are reported. We studied the correlation of BMI and metabolite levels with all the samples together and also dividing samples depending on their clinical status.

### Multivariate analysis

Principal Component Analysis (PCA), Partial Least Squares-Discriminant Analysis (PLS-DA) and Orthogonal Partial Least Squares (OPLS) were performed for each pairwise comparison using SIMCA-P v12.0.1.0 software (Umetrics *AB*).

### Metabolites mapping into cellular metabolic pathways and identification of primary enzymes associated with their metabolism

Metabolic pathways were determined with MetScape v3.1.2 application, running under Cytoscape v3.5.0 software, linking them to KEGG Pathway database (http://www.genome.jp/kegg/pathway.html). Primary enzymes involved in the metabolism of the metabolite of interest, and their corresponding coding genes were retrieved from KEGG (*http://www.genome.jp/kegg/compound/*) and HMDB (*http://www.hmdb.ca/*) databases, using dbWalk utility on bioDBnet database searching online utility and specifying “9606” (Homo sapiens) Taxon ID on Organism box (https://biodbnet-abcc.ncifcrf.gov/db/dbWalk.php), with the following paths:

- For KEGG compounds, we started with enzyme EC code:

EC Number->UniProt Accession->UniProt Entry Name->KEGG Gene ID->Gene ID->Gene Symbol->Gene ID->GenBank Nucleotide Accession.

- For HMDB compounds, we started with the name present on HMDB database:

HMDB Metabolite->HMDB Enzyme -> UniProt Entry Name->Gene Symbol->Gene ID->GenBank Nucleotide Accession.

For each metabolite included in this step, we reported:

- For KEGG compounds, the related enzymes EC number, UniProt Accession, UniProt Entry Name, KEGG Gene ID, Gene Symbol, GeneID and the GenBank Nucleotide Accession for the corresponding transcripts.
- For HMDB compounds, the HMDB enzyme Gene Symbol, Gene Symbol, Gene ID and GenBank Nucleotide Accession for the corresponding transcripts.

Database normalisation: all the datasets used for the data mining analysis were downloaded from GEO or TCGA, and subjected to background correction, log_2_ transformation and quartile normalisation as reported [20,21]. In the case of using a pre-processed dataset, this normalisation was reviewed and corrected if required. For normal *vs*. PCa comparisons, a two-tailed *t*-test is performed in order to indicate if the observed differences between the groups are significant. For tumour progression analysis, an ANOVA test was performed in order to evaluate if the observed differences of gene expression levels between the groups were significant. DFS analysis was performed using Taylor and TCGA datasets. In both cases, the patients were stratified by quartiles based on the expression of the gene of interest, Kaplan-Meier Estimator was used in order to estimate the survival function from different groups of patients while a Log-Rank test is calculated to check the significance between the curves. In the case of Taylor dataset, the analysis was performed using the average signal from all the transcripts of a gene.

## Results

**Figure 1.**
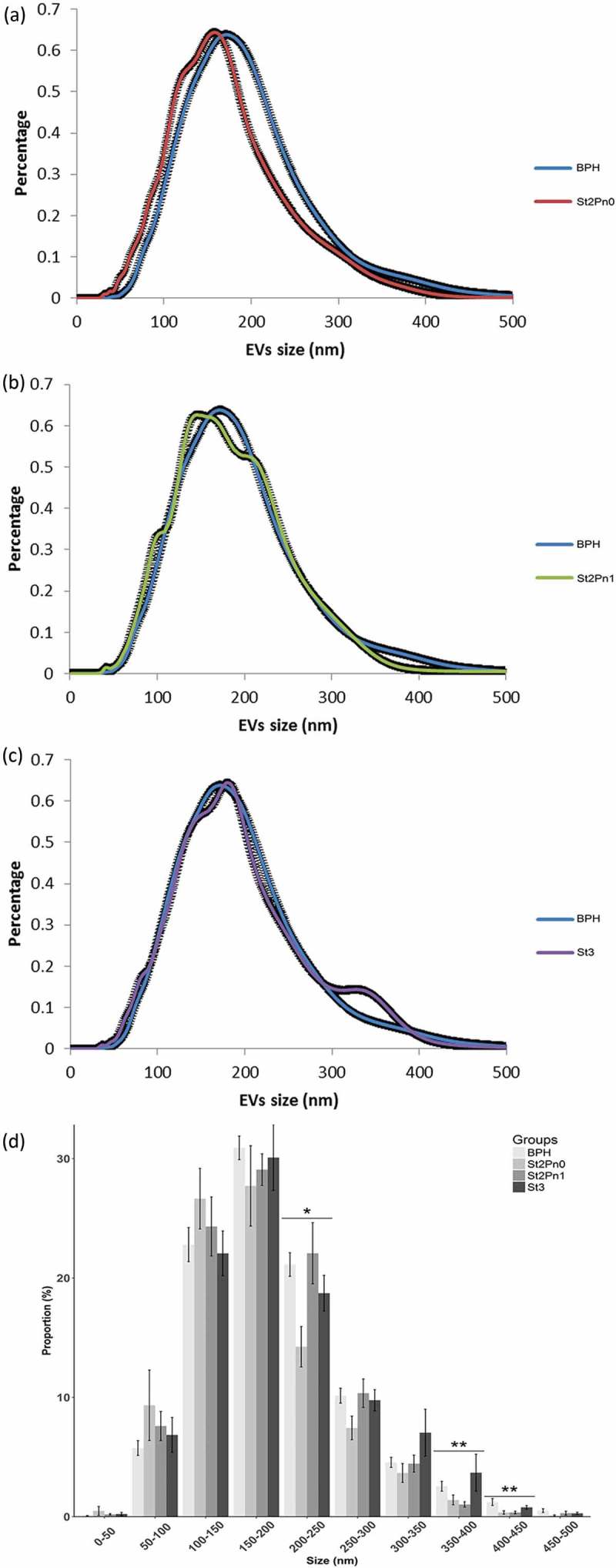
Size distribution of urinary EVs isolated from the BPH and PCa groups. Pairwise comparison BPH vs PCa stage 2 without perineural invasion (a), pairwise comparison BPH vs PCa stage 2 with perineural invasion (b) and pairwise comparison BPH vs PCa stage 3 (c). Size distribution of the particles isolated from each patient, including SEM error bars (d). Number of samples: BPH (*n* = 14), Stg.2 Pn0 (*n* = 6), Stg.2 Pn1 (*n* = 10) and Stg.3 (*n* = 13), all of them analysed in duplicate. Kruskal-Wallis Rank Sum test was applied to study the significance of the sizes distribution differences (*p < 0.05 and **p < 0.01).

Urine samples were collected from patients with BPH (*n* = 14) and PCa (*n* = 31) with different pathological characteristics (Table 1). In order to avoid any chemical alteration of the vesicles that could interfere with the metabolomics analysis, we decided to preserve uromodulin status of the samples by avoiding the use of high-salt concentration or reducing agents. After initial clearing at low centrifugation and ultrafiltration, small EVs (exosomes, small microvesicles and apoptotic blebs) were isolated by differential ultracentrifugation as described in [16]. Cryo-electron microscopy revealed the presence of vesicles in the preparations (Supplementary Figure 1A). Western-blot analysis showed that while we could not detect mitochondria (COX IV) or endoplasmic reticulum (GRP78) proteins, we could detect exosomal markers (CD10, CD63, CD9, Flotillin and CD26), and also some uromodulin (THP) (Supplementary Figure 1B). As previously, we found a high inter-individual variability in the abundance of these proteins [16,22]. In agreement with previous results [16], physical characterisation by NTA analysis of the isolated material revealed significant differences in the size distribution of particles isolated from PCa and BPH samples (Figure 1). Interestingly, the size of the particles increased with the stage of the PCa, thus, the major difference was observed between BPH and PCa stage 3 (Figure 1). A significant higher abundance of particles bigger than 350 nm were observed in samples from PCa stage 3 (Figure 1(d)). The mean concentration of particles per mL for all samples was 8.60 ± 1.19 × 10^10^ EVs/mL. No differences were found for the concentrations of EVs/mL between different groups (BPH, PCa stage 2 Pn0, stage 2 Pn1 and stage 3).

After this initial characterisation, metabolites present in the urinary EV preparations were extracted using different methodologies in order to cover a wide range of molecules with different chemical nature (see *Methods* section). We were able to detect 248 metabolites (Supplementary Table 1) including amino acids, vitamins, nucleosides, as well as different lipid species. Considering all the samples, metabolites with more than 70% of missing values were eliminated from the analysis with the exception of PC(14:0/20:4), PC(0:0/20:3) and TG(56:8) because most of missing values occur mainly in one of the two groups (PCa or BPH). Afterwards, we performed three different statistical analysis comparing BPH and PCa groups, as well as, the association to tumour stage and perineural invasion.

## Metabolites differentially altered between BPH and pca

**Figure 2.**
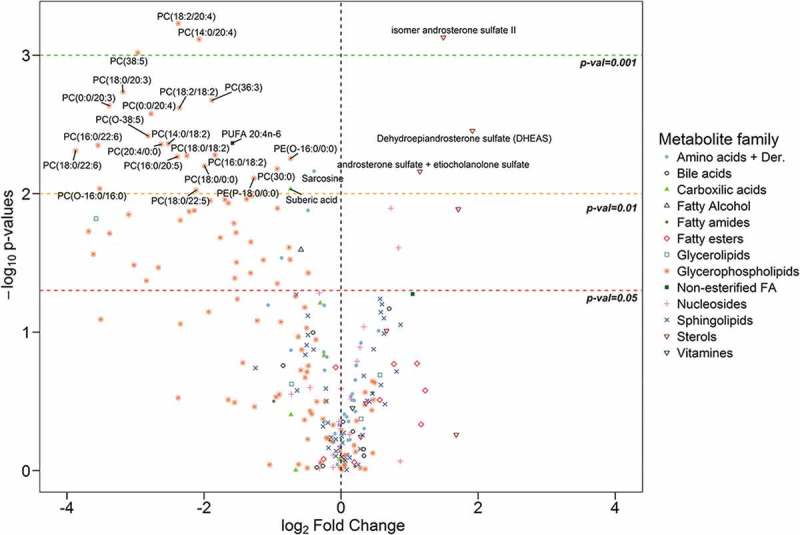
Volcano plot for BPH (*n* = 14) *vs* PCa (*n* = 31). Positive fold change indicates an increase of the metabolite in PCa, while a negative value indicates that the levels are reduced in PCa. Dots shape and colour depend on metabolite families.

**Figure 3.**
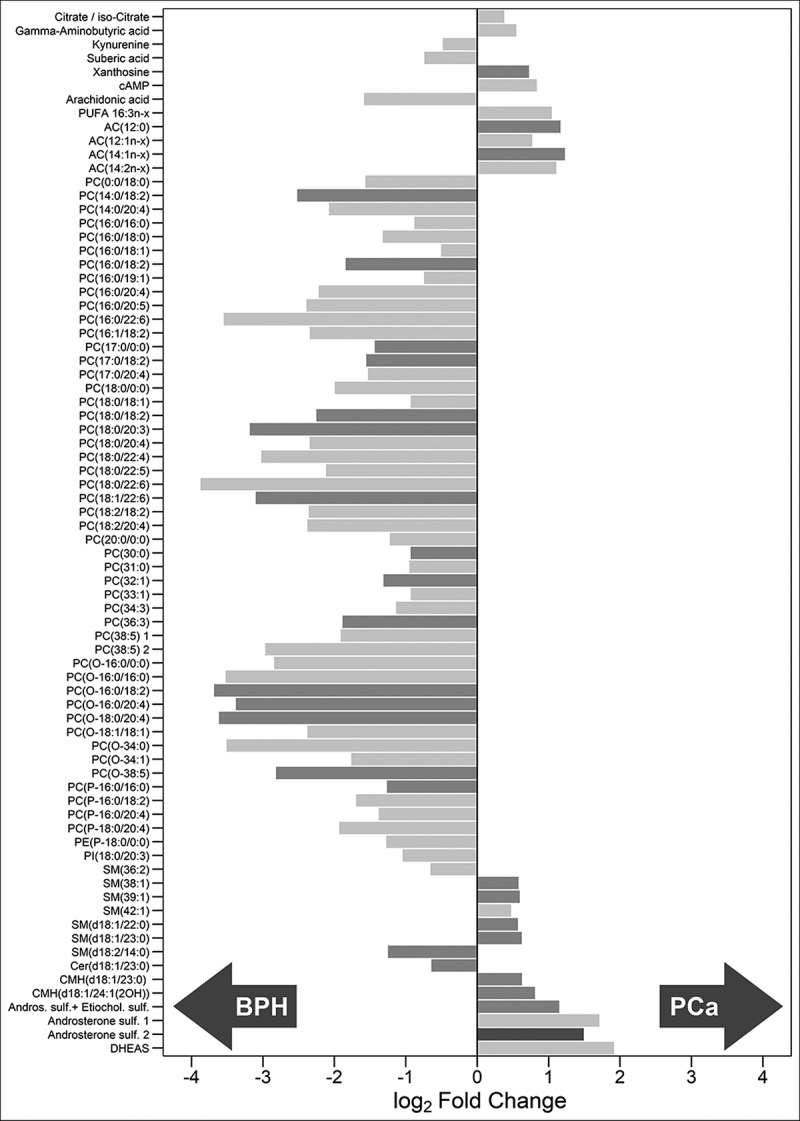
Metabolites associated to urinary EVs differentially expressed between BPH (*n* = 14) and PCa samples (*n* = 31). Bars have been coloured depending on the significance of the differences, being lighter gray for the *p*-values between 0.05 and 0.01, medium gray for *p*-values between 0.01 and 0.001 and darker gray for *p*-values lower than 0.001.

Univariate analysis revealed that 76 out of 248 metabolites showed statistically significant differences between EVs from PCa and BPH patients. These metabolites were distributed along most chemical families analysed, although there was a predominance of phosphatidylcolines (PC), fatty acid esters (acyl carnitines) and sterols (Figures 2, 3 and Supplementary Figure 2). Whereas higher abundance of PC was observed in BPH samples, acyl carnitines and sterols were more abundant in PCa samples (Figures 2 and 3). In addition, carboxylic acids and glycerolipids were slightly decreased, and vitamins were increased in PCa EVs. The other families of metabolites including amino acids, bile acids, nucleosides, sphingolipids, phosphatidylethanolamines (PE) contained both increased and decreased metabolites (Figure 3). Interestingly, the abundance of ceramides with short carbon number in their acyl chains were increased in PCa samples, while ceramides with long carbon number (>23) in their acyl chains were reduced in PCa EVs. This pattern was not present in other sphingolipids families. In the non-esterified fatty acid family, the abundance of arachidonic acid (20:4n-6) was decreased in PCa samples, while other polyunsaturated fatty acid with shorter carbon chain (16:3n-x) was significantly increased in the PCa group (Figure 3).

**Figure 4.**
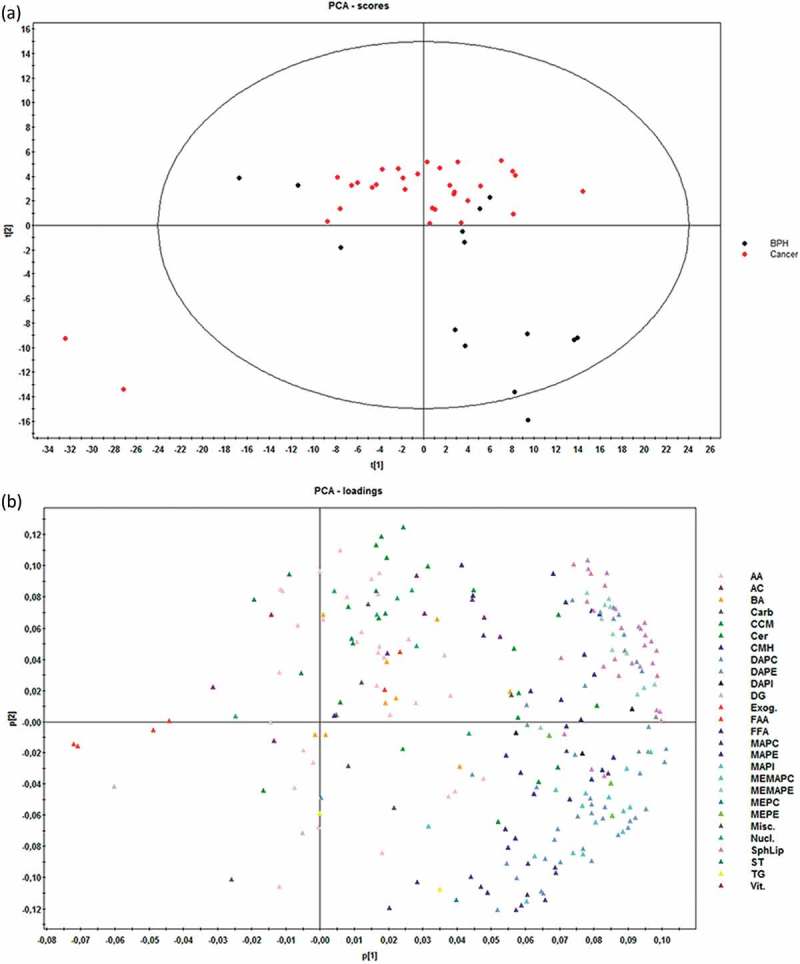
Score (a) and loadings (b) plots of PCA model for the comparison PCa (*n* = 31) *vs* BHP (*n* = 14). Dots in score plot (A) have been coloured depending on its group (PCa or BHP). Markers in loadings plot (B) have been coloured depending on metabolite family. AA (amino acids), AC (acyl carnitines), BA (bile acids), Carb (carboxylic acids), CCM (derivative carboxilic acids), Cer (ceramides), CMH (monohexosylceramides), DAPC (diacylglycerophosphocholines), DAPE (diacylglycerophosphoethanolamines), DAPI (diacylglycerophosphoinositol), DG (diacylglycerols), Exog. (exogenous), FAA (fatty amides), FFA (non-esterified fatty acids), MAPC (1-monoacylglycerophosphocholine), MAPE (monoacylglycerophosphoethanolamine), MAPI (monoacylglycerophosphoinositol), MEMAPC (1-ether, 2-acylglycerophosphocholine), MEMAPE (1-ether, 2-acylglycerophosphoethanolamine), MEPC (1-monoetherglycerophosphocholine), MEPE (1-monoetherglycerophosphoethanolamine). See more details of the nomenclature in supplemental material.

Multivariate analysis by principal component analysis (PCA) did not show a perfect separation of the two groups, although PCa EV samples tended to aggregate all together, whereas BPH samples were more disperse (Figure 4(a)). Statistics of the model indicate low degree of fit (2n component R2X = 0.49) and also low predictability (2n component Q2X = 0.37). The PCA loadings plot (Figure 4(b)) indicated that the differences between PCa and BPH samples were explained mainly by different subfamilies of glycerophospholipids, confirming what was identified with the univariate analysis.

## Metabolites differentially altered between PCa stage 2 and stage 3

**Figure 5.**
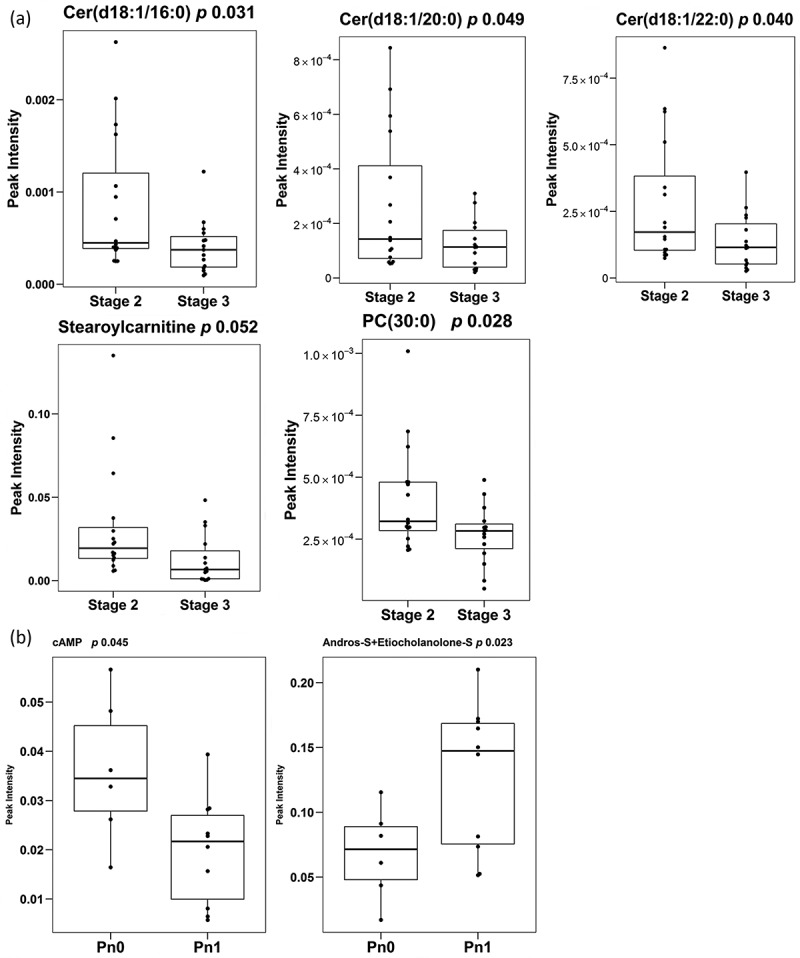
Differentially-expressed metabolites. Box-plots of differentially expressed metabolites between PCa stages (A) (stage 2 *n* = 16 and stage 3 *n* = 15) and of differentially expressed metabolites between the presence and absence of perineural invasion (B) (Pn0 *n* = 6 and Pn1 *n* = 10). Significance is indicated next to metabolite name.

PCa stage is a pathological sign of disease aggressiveness [1]. In an attempt to identify potential biomarkers to discriminate between different stages of PCa, we performed univariate analysis comparing the PCa stage 2 and stage 3 subgroups. We identified 5 metabolites that showed significant differences between the two groups (Figure 5(a)). These metabolites were three ceramides, Cer(d18:1/16:0), Cer(d18:1/20:0), Cer(d18:1/22:0) one glycerophospholipid PC(30:0) [which is a combination of the isomers PC(16:0/14:0) and PC(14:0/16:0)] and one acyl carnitine, stearoylcarnitine [AC(18:0)]. In addition, we also observed a non-significant trend in other metabolite families. Thus, fatty esters, glycerolipids (both diacylglycerols and triacylglycerols), fatty amides, vitamins and 1-monoetherglycerophosphocholines showed an increase in their abundance in the PCa stage 3 group (Supplementary Table 1). In contrast, the levels of most of the metabolites belonging to the sphingolipids family including ceramides, monohexosylceramides and sphingomyelins, as well as fatty alcohols, some glycerophospholipids subgroups and nucleosides were reduced in stage 3. In this comparison, unsupervised multivariate analysis could not achieve any separation between different PCa stages, and although supervised PLS-DA analysis was able to discriminate (R2X 0.47, Q2X 0.07), its loadings plot showed that the major influence in the separation corresponded to the aforementioned five metabolites (*data not shown*) detected in the univariate analysis.

## Metabolites differentially altered between PCa stage 2 perineural invasion: Pn1 *vs* Pn0

Perineural invasion in PCa has been associated to prostate cancer prognosis [23]. Although a limited number of samples were available, we also attempted to identify metabolites tentatively associated to this pathological feature. By univariate analysis, we detected significant lower abundance of cyclic AMP (cAMP) and higher abundance of the combination of isomers androsterone sulphate and etiocholanolone sulphate in the EV samples obtained from PCa patients with perineural invasion (Figure 5(b)). In addition, although not significant, three bile acids showed lower levels in samples with perineural invasion (Supplementary Table 1). Unsupervised multivariate analysis was not able to separate the two groups of samples, but we could achieve this separation with PLS-DA test (R2X 0.40, Q2X 0.58) (*data not show*n).

## Correlation analysis of metabolic profiling with body mass index (BMI)

When studying circulating metabolites, the systemic metabolic state can be a critical contributing factor that can influence the results of the analysis. Obesogenic diets and obesity impact on biofluid metabolite concentration, and can also have a central effect on tumour tissues [24] by altering their biological features. Therefore, we considered evaluating the changes in urine EV metabolites that were associated to the body mass index (BMI). Samples were divided into three groups, corresponding to their calculated BMI: lean (<25), overweight (>25 and <30) and obese (>30). Taking into account all the samples independently of their BPH or PCa classification, no significant correlation was found between BMI and any of the 248 metabolites analysed in this study. Afterwards, we explored if some metabolites were correlating with BMI inside different groups. In the lean BMI group, some sterol-related metabolites including isomer pregn-5-ene-3,20-diol sulphate and isomer androsterone sulphate showed significant positive correlations with *rho* values of 0.72 and 0.60, respectively (Table 2). On the contrary, diacylglycerol DG(36:3), PC(18:2/00) and triglyceride TG(56:3) showed significant negative correlation with *rho* values of −0.71, −0.69 and −0.67, respectively (Table 2). In the case of the overweight BMI group, significant positive correlation was found with the exogenous metabolite, hydroxyphenyllactic acid (ρ 0.69). Sphingomyelin SM(43:1) showed significant negative correlation (ρ −0.67) with BMI values (Table 2). In the obese group, we observed a high degree of correlation of some metabolites with the BMI values. Thus, acyl carnitine AC(8:0) (ρ 0.94) and arginine (ρ 0.85) showed significant positive correlation, while 13 sphingomyelins, 8 phosphatidylethanolamines and the polyunsaturated fatty acid (16:3n-3) showed negative correlations with *rho* values ranging between −0.95 to −0.78) (Table 2). Finally, we evaluated if any of the metabolites correlated with BMI considering only the PCa group. Inside this group the highest positive correlations were found for taurocholic acid and dodecanoylcarnitine, AC(12:0), with *rho* values of 0.51 and 0.38, respectively.

**Table 2.** Correlation analysis of metabolites and BMI.

	Metabolite	Class	Correlation (*ρ*)	*p-value*
Lean	Isomer pregn-5-ene-3,20-diol sulphate	Sterol	0.72	0.003
	Isomer androsterone sulphate	Sterol	0.60	0.011
	Taurodeoxycholic acid	Bile acid	0.59	0.03
	Malate	Carboxylic acid (d)	0.56	0.025
	Arginine	Amino acid	0.53	0.027
	Glycine	Amino acid	−0.56	0.021
	TG(18:1 + 20:1 + 18:1)	Glycerolipid	−0.67	0.006
	PC(18:2/0:0)	Glycerophospholipid	−0.69	0.007
	DG(36:3)	Glycerolipid	−0.71	0.008
Overweight	Hydroxyphenyllactic acid	Benzyl alcohol (d)	0.69	0.001
	L-citruline	Amino acid (d)	0.59	0.008
	Vitamin B5	Vitamin	0.52	0.024
	Proline	Amino acid	0.50	0.030
	DG(34:1)	Glycerolipid	0.49	0.035
	4-Pyridoxic acid	Pyridine (d)	0.49	0.036
	PC(O-16:0/20:4)	Glycerophospholipid	0.47	0.042
	PE(18:0/18:1)	Glycerophospholipid	−0.47	0.046
	SM(d18:1/17:0)	Sphinomyelin	−0.48	0.038
	Stearoylcarnitine	Acyl caritine	−0.49	0.037
	PE(P-18:0/18:1)	Glycerophospholipid	−0.50	0.030
	PE(16:0/18:2)	Glycerophospholipid	−0.51	0.027
	PE(0:0/20:3)	Glycerophospholipid	−0.51	0.027
	PE(P-16:0/18:2)	Glycerophospholipid	−0.53	0.021
	Alpha-Ketoglutarate	Keto-acids (d)	−0.54	0.028
	PE(18:1/18:2)	Glycerophospholipid	−0.55	0.017
	PE(P-18:0/18:2)	Glycerophospholipid	−0.56	0.013
	AC(12:1n-x)	Fatty esters	−0.57	0.014
	PE(18:2/18:2)	Glycerophospholipid	−0.57	0.016
	SM(43:1)	Sphingomyelin	−067	0.003
Obese	L-Octanoylcarnitine	Acyl carnitine	0.94	0.017
	Arginine	Amino acid	0.86	0.024
	PC(O-16:0/18:2)	Glycerophospholipid	0.83	0.058
	Acylcarnitine(8:1n-x)	Acyl carnitine	0.75	0.066
	Deoxycholic acid	Bile acid	0.75	0.066
	PI(18:0/20:4)	Glycerophospholipid	−0.75	0.066
	PE(20:5/16:0)	Glycerophospholipid	−0.75	0.066
	L-Homoserine	Amino acid	−0.75	0.066
	Isoleucine	Amino acid	−0.75	0.066
	SM(43:1)	Sphingomyelin	−0.79	0.048
	SM(d18:1/24:1) + SM(d18:2/24:0)	Sphingomyelin	−0.79	0.048
	SM(d18:1/17:0)	Sphingomyelin	−0.79	0.048
	SM(33:1)	Sphingomyelin	−0.79	0.048
	PE(P-18:0/22:5) + PE(P-20:1/20:4)	Glycerophospholipid	−0.79	0.048
	PUFA (16:3n-x)	Fatty acid	−0.79	0.048
	PE(20:4/18:2)	Glycerophospholipid	−0.79	0.048
	SM(32:1)	Sphingomyelin	−0.82	0.034
	PE(P-16:0/20:4)	Glycerophospholipid	−0.82	0.034
	PE(0:0/22:4)	Glycerophospholipid	−0.82	0.034
	SM(d18:2/22:0)	Sphingomyelin	−0.86	0.024
	SM(d18:1/22:0)	Sphingomyelin	−0.86	0.024
	SM(d18:1/18:0)	Sphingomyelin	−0.86	0.024
	SM(d18:1/16:0)	Sphingomyelin	−0.86	0.024
	PE(18:0/20:4)	Glycerophospholipid	−0.86	0.024
	PE(18:1e/22:4)	Glycerophospholipid	−0.88	0.008
	SM(d18:2/20:0)	Sphingomyelin	−0.89	0.012
	PE(16:0/22:6)	Glycerophospholipid	−0.89	0.012
	SM(42:1)	Sphingomyelin	−0.93	0.007
	SM(d16:1/24:1)	Sphingomyelin	−0.93	0.007
	PE(16:0/20:4)	Glycerophospholipid	−0.93	0.007
	SM(38:1)	Sphingomyelin	−0.96	0.003

## Correlation analysis of metabolic profiling with PSA in the PCa group

PSA is the current gold standard non-invasive prognostic marker for PCa while its diagnostic potential remains controversial [25]. We performed a correlation analysis between urinary EV metabolites and the PSA values determined in our cohort of PCa samples. We only observed a significant positive correlation (*rho* value 0.88) of phosphatidylcoline PC(0:0/20:3), and at less extent (*rho* value 0.48) of the primary fatty amide (20:2n-x).

## Analysis of enzymes-associated to metabolites differentially expressed between PCa and BPH

**Figure 6.**
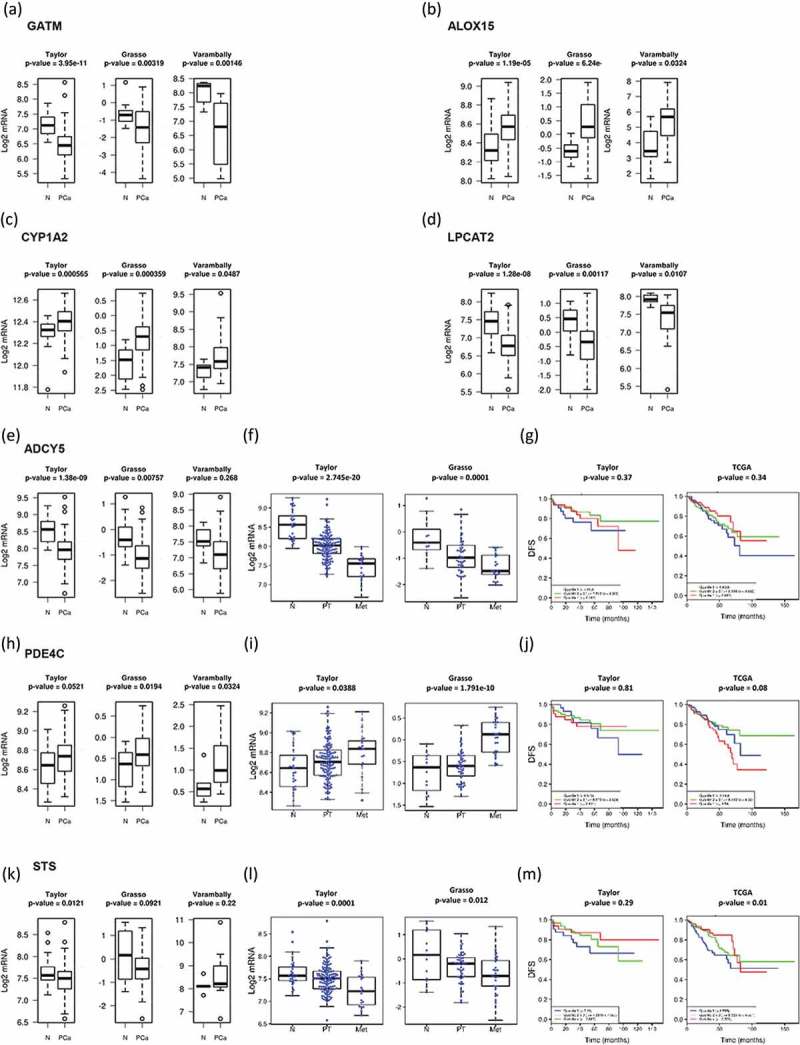
Gene-enrichment analysis. *In-silico* transcriptomics analysis of enzymes directly involved in the metabolism of metabolites differentially expresed between PCa and BPH samples.

We have recently shown that metabolic alterations in PCa are frequently associated to changes in the expression of key enzymes [21]. To better understand the cancer cell autonomous nature of the metabolite changes observed in urine EVs from PCa patients, we mapped the 76 altered urinary-EV-metabolites into cellular pathways by using MetScape v3.1.2 [26]. We identified several pathways that could be affected in PCa including steroid hormone biosynthesis and metabolism, leukotriene and prostaglandin metabolisms, linoleate and purine metabolisms, glycerophospholipid metabolism, TCA and urea cycle, and tryptophan metabolism. We identified the primary enzymes involved in the metabolism of each of the 76 differentially expressed metabolites between BPH and PCa, by using KEGG or HMDB database (see *Methods*). A complete list of primary enzymes is supplied as Supplementary Table 2. Next, we took advantage of publicly available prostate cancer transcriptomes and we queried the expression of the 149 enzymes in PCa. We searched for enzymes which expression changes in PCa would fit the metabolite abundance observed in urine EVs. From these gene list, we identified 7 genes with the expression changes (Figure 6) that were concordant with the observed changes in urine EV metabolite abundance among the groups analysed. We found gamma-aminobutyric acid (GABA) increased in PCa urine EVs (Figure 3) which was consistent with a reduction in the expression of Glycine Amidinotransferase (GATM- use GABA as substrate for creatine synthesis) (Figure 6(a)). Arachidonic acid abundance was also altered in urine EV samples, being reduced in PCa patients compared with BPH (Figure 3). This fatty acid is the product of phospholipase A2 and it is relevant for the synthesis of pro-inflammatory metabolites by lipooxygenases. Interestingly, we found that the expression of two enzymes (ALOX15 and CYP1A2), that can catabolise arachidonic acid, was increased in PCa tissue (Figure 6(b,c). Our metabolomics analysis also showed a consistent decrease in phosphatidylcholine. This could be explained by decreased synthesis of the phospholipid or elevated catabolism. When browsing the expression of PC synthesis and degrading enzymes, we found a reduction in the expression of Lysophosphatidylcholine Acyltransferase 2 (LPCAT2) (Figure 6(d)), which transforms lysoPC into PC, and could provide an explanation for the reduction in PC abundance.

Two urine EV metabolites were associated to increased perineural invasion in PCa. On the one hand, we found a decrease in cAMP abundance in EV obtained patients with perineural invasion. The transcriptional analysis revealed changes in the expression of enzymes regulating cAMP synthesis and degradation that were associated to the aggressiveness of the disease. The expression of adenylate cyclase 5 (ADCY5) was reduced in PCa (Figure 6(e–g))), and a further significant reduction was observed from primary tumours to metastasis. In contrast, the inverse expression pattern (elevation in PCa and further increase from primary tumours to metastasis) was detected in the cAMP degrading enzyme PDE4C (Figure 6(h–j)). In none of these cAMP metabolising enzymes we could find an association to altered disease-free survival (Figure 6(g,j)).

On the other hand, the steroid biosynthesis-related metabolites were among the most elevated in PCa urine EVs, and associated with increased perineural invasion. Interestingly, the three metabolites significantly altered were sulphated steroids in the final steps of androgen synthesis. Whereas these metabolites are found at detectable levels in circulation produced by the adrenal gland, we evaluated whether enzymes regulating their synthesis or degradation could be altered in PCa tissue. Strikingly, we found that the expression steroid sulfatase (STS), which would remove the sulphate group in androsterone sulphate and DHEAS, was decreased in PCa, and this reduction was associated to metastatic disease and reduced disease-free survival in one out of two datasets (Figure 6(k–m).

## Discussion

EVs are produced by normal and cancerous cells and harbour molecular features of their cells of origin [27]. This encapsulated material can exert biological and metabolic functions [4,14,15,28], which makes them entities of tremendous interest in cancer biology, both at the level of biomarker discovery and mechanistic. Urine contains EVs from different parts of the urinary track including kidney and bladder what has awaked great interest to identify biomarkers affecting these organs. In addition, the anatomic proximity of urine to the prostate gland and the already shown presence of tumour cells in the urine sediment [29,30] support also the development of potential non-invasive diagnoses of PCa using urine-based markers. In agreement with our previous results, we find differences in the size distribution of the urinary EVs between PCa and BPH [16], which we now report to be associated to disease stage (Figure 1). Our data show that urine from advanced PCa patients contains a higher proportion of large EVs than BPH patients. Given that our EV isolation procedure (filtration through 0.22 microns, and ultracentrifugation at 10,000 × g) removed most of the large EVs from the sample, and enrich in small EVs (mostly exosomes and small microvesicles), this difference could be underestimated in our samples. Importantly, in agreement with our result, it has already been reported that prostate cancer cells release large EVs named oncosomes with a size between 1 and 10 microns [31] that contain a distinct protein cargo [32]. They have also been detected in circulation in models of PCa and shown that their abundance correlate with tumoral progression [31]. Although, our studies have been focused in the smaller EVs, it is interesting that we have also observed this size effect.

In a recent targeted lipidomics analysis of urinary EVs from healthy and PCa urine samples [13], the authors analysed 107 lipid species and found that 9 of them were significantly different between the two groups. Unlike this study, we have focused ours in the comparison between PCa and BPH, in an attempt to provide specific biomarkers to discriminate the two pathological conditions, and contribute to earlier diagnosis, and reduce secondary effects of unnecessary biopsies, so both studies can be considered complementary in terms of sample groups. Both studies are also complementary in the metabolites that they analyse because different metabolite extraction method and chromatographic procedures were used.

We report changes in the urine EV metabolome at both structural and cargo levels. The composition of the urine EVs analysed in this study varies in the abundance of phosphatidylcholine species that are major constituents of membranes. In particular we found reduced abundance of PCs in the EVs from PCa samples, in agreement with previously reported by Puhka and coworkers [33]. This result along with studies reporting increased abundance of PC in PCa tissue [34] could suggest that less PC-containing structures, like membrane vesicles are secreted to the extracellular environment. In addition, to the PC content, we found additional metabolites from different chemical nature differentially expressed in EVs from PCa and BPH samples that could be considered candidate biomarker for PCa including as candidate acyl carnitines, sphingomyelins, and steroids. Although more research is granted, our results indicate that a bias in EV size and membrane composition could harbour diagnostic potential in PCa.

Apart of the potential biomarker value of the identified metabolites, they are also valuable to indicate possible metabolic alterations occurring in PCa. We found reduced levels in PCa urine EVs of arachidonic acid, the precursor of eicosanoids and prostaglandins that are important proliferative and inflammatory modulators. Interestingly, it has been also reported that arachidonic acid level is lower in prostatic tissue from PCa patients [35]. In agreement with the reduction of the substrate arachidonic acid in PCa, it has been found that the level of their products (12-and 20-HETE, and PGE2) are higher in the tissue [36,37] and also in urine [38]. These studies along with many others have already shown that the metabolism of arachidonic acid and their products plays an important role in PCa development, and in fact, represents an important therapeutics target (reviewed in [39]). Importantly, our work suggests that the analysis of this metabolite in EVs isolated from urine samples may be used to evaluate in a non-invasive manner what is occurring in prostatic tissue itself in the context of PCa.

We observed changes in the abundance of metabolites that are carried within the EVs and are a potential cargo in PCa. It is worth mentioning that intermediary metabolites of androgen synthesis were among the most elevated in PCa urine EVs. Moreover, changes in the abundance of these steroids, together with cAMP, were significantly associated to perineural invasion. These results uncover the potential of unbiased urine EV analysis to elucidate novel signalling and metabolic alterations underlying PCa biology. Androgen signalling is among the predominant stimuli supporting PCa growth and the most successful therapeutic approaches have derived from its targeting [40], since prostate tumours frequently remain androgen dependent even at late-stage [41]. We have detected 3beta-hydroxyandors-5-en-17-one-3-sulphate (dehydroepiandrosterone sulphate, DHEAS) in urinary EVs, and its level was significantly elevated in PCa samples. This metabolite, along with estrone sulphate, is one of the main precursor for steroid hormones including androgens. There are many reports showing that steroid-related metabolites and enzymes are important modulators of PCa progression [42]. There are four different genes coding for enzymes that were related to this metabolite: STS, SULT1B1, SULT2B1 and SULT2A1. The fact that urine EVs from PCa patients contain androgen-related metabolites is suggestive of the relevance of this biosynthetic pathway in the disease and the potential role of EVs in providing androgen signalling to neighbour or distal cells. Indeed, expression of STS was reduced in PCa and associated to disease progression, hence providing a feasible explanation for the increase in sulfated steroids. Interestingly, urinary EVs could be used to monitoring androgen metabolism in a non-invasive manner.

Together with the aforementioned metabolites associated to perineural invasion, we also identified molecules that exhibited differential abundance in high grade tumours. Five metabolites were differentially abundant between pathological stage 2 and stage 3 PCa, and more than half of them were ceramide species. Ceramides are signalling molecules that can regulate various aspects of cancer cell biology, including proliferation, survival and cell death [43]. The selective decrease of ceramides in association with disease aggressiveness provides an exciting perspective of how this family of metabolites could exert cell and non-cell autonomous functions to limit the progression of PCa.

It is worth noting that sarcosine has been proposed also as a PCa biomarker [11]. The urine level of this metabolite was increased in men with metastatic PCa [44]. However, its utility as a potential diagnostic tool is unclear, as its validation as a biomarker has failed in several studies (reviewed in [11,45]). Interestingly, we have detected sarcosine in urinary EVs, and although not significant (*p = *0.09), its level was decreased in PCa samples.

Recent molecular and metabolic profiling of PCa also identifies lipid metabolism as a key pathway that undergoes metabolic reprogramming [46,47]. These changes include an upregulation metabolites involved in *de novo* lipid biosynthesis [48] and fatty acid β-oxidation [49]. As consequence, it has been shown the accumulation in the prostatic tissue of acyl carnitines, which are intermediates of fatty acid oxidation [50]. In agreement with this alteration, we found increased levels of acyl carnitines in the urinary EVs from PCa patients. This association of differential levels of carnitines on PCa EVs with a metabolic shifting towards β-oxidation of fatty acids has already been proposed by Puhka and coworkers [33].

In summary, in this work we report several metabolites associated to urinary EVs, many of them exhibiting differential abundance between BPH and PCa, and mirroring some of the alterations described in PCa.
