# Supplementary material for: Metabolic alterations in urine extracellular vesicles are associated to prostate cancer pathogenesis and progression
Source: J Extracell Vesicles. 2018 May 7;7(1):1470442. doi: 10.1080/20013078.2018.1470442 (PMC5944373; doi:10.1080/20013078.2018.1470442)
Supplement: Supplemental_files.zip [file ZJEV_A_1470442_SM6791.zip › Supplemental files/Supplementary Nomenclature.docx]

**Supplementary material**

**Lipid nomenclature**

Lipid nomenclature and classification follows the LIPID MAPS convention ([www.lipidmaps.org](http://www.lipidmaps.org)).

**Fatty acyl species: fatty acids (FA), acyl carnitines (AC), and N-acylethanolamines (NAE)**

The nomenclature *A:B n-C* is used for fatty acyl species, where *A* is the number of carbon atoms, *B* is the number of double bonds in the fatty acid chains or the level of chain desaturation, and *C* refers to the position of the first double bond from the omega end. The omega-reference system was selected to indicate double bond position since the physiological properties of unsaturated fatty acids largely depend on the position of the first unsaturation relative to the end position, existing physiological differences between omega - 3 and omega - 6 fatty acids. If *C* is designated as “x” in the *C*, it indicates the unknown position the double bounds, as reference standards for full identification were not available.

**Oxidized fatty acids**

Oxidized fatty acids derived from the metabolism of polyunsaturated fatty acids are indicated as follows: Hydroxy-octadecadienoic acids (HODE), Oxo-octadecadienoic acids (OxoODE), Dihydroxy-octadecadenoic acids (DiHOME), Trihydroxy-octadecadenoic acids (TriHOME), and Hydroxy-eicosatetraenoic acids (HETE).

**Glycerolipids species: monoacylglycerides (MAG), diacylglycerides (DAG) and triacylglycerides (TAG)**

The *A:B* nomenclature is used for glycerolipids species, where *A* is the number of carbon atoms and *B* is the number of double bonds considering all the acyl chains esterified to glycerol. The position of the double bonds in the acyl chains is not considered, as well as the position of the acyl chains.

**Glycerophospholipids: glycerophosphocholines (PC), glycerophosphoethanolamines (PE), glycerophosphoinositols (PI) as diacyl, monoacyl, monoether or monoether-monoacyl species**

The *A:B/C:D* nomenclature is used, where *A:B* and *C:D* refer to the number of carbon atoms and number of double bonds contained in the sn-1 and sn-2 side chains, respectively. *X:Y* nomenclature (where *X* = *A*+*C* and *Y* = *B*+*D*) is used where evidence was found for the contribution of multiple species to a single chromatographic peak. Lysoglycerophospholipids are indicated as *A:B/0:0* or *0:0/C:D* for the fatty acid linkage at the *sn*-1 or *sn*-2 position of the glycerol backbone, respectively. The position of the double bonds in the acyl chains is not considered.

An additional classification of the ether-glycerophospholipids is provided, differentiating between 1-alkyl ether (plasmanyl) and 1-(1Z-alkenyl) ether, also named plasmalogens, vinyl ether or plasmenyl lipids. 1-alkyl ether-glycerophospholipids are indicated as *O*-*A:B/C:D*, while vinyl ether-glycerophospholipids are indicated as *P*-*A:B/C:D*, where *A:B* and *C:D* refer to the number of carbon atoms and number of double bonds contained in the sn-1 and sn-2 side chains. The suffix “e” indicates the presence of an ether linked substituent if the ether-glycerophospholipids cannot be classified as 1-alkyl or vinyl ether-glycerophospholipid.

**Sphingolipids species: sphingomyelins (SM), ceramides (Cer), and monohexosylceramides (CMH)**

For sphingolipids, *sA:B/C:D* nomenclature is used, where *sA:B* represents the sphingoid base: s18:1, sphingosine; s18:2, sphingadiene; s18:0, sphinganine. *C:D* indicates the number of carbon atoms *C*, and double bonds *D*, contained in the N-linked fatty acid. The position of the double bonds in the acyl chains is not considered.

**Cholesteryl esters (ChoE)**

The *A:B* nomenclature is used, where *A* is the number of carbon atoms and *B* is the number of double bonds in the acyl chains esterified to the cholesterol. The position of the double bonds in the acyl chains is not considered.
