# Supplementary material for: Metabolic alterations in urine extracellular vesicles are associated to prostate cancer pathogenesis and progression
Source: J Extracell Vesicles. 2018 May 7;7(1):1470442. doi: 10.1080/20013078.2018.1470442 (PMC5944373; doi:10.1080/20013078.2018.1470442)
Supplement: Supplemental_files.zip [file ZJEV_A_1470442_SM6791.zip › Supplemental files/SUPPLEMENTARY_TABLE_1.docx]

**Supplementary Table 1.** Values of Fold Change (FC) and p-values the metabolites included in the study. We also indicate the number of samples of each group (PCa and BPH) in which metabolites were detected. * indicates those metabolites that were removed from the statistical analysis because they had less than 70% of values for some of the two comparisons. Fold change and p-value are indicated with italic red 0.

|  |  |  | PCa *vs* BPH | | | Stg. 3 *vs* stg. 2 | | Pn1 *vs* Pn0 | |
| --- | --- | --- | --- | --- | --- | --- | --- | --- | --- |
|  | **PCa (n)** | **BPH (n)** | | **FC** | ***p*** | **FC** | ***p*** | **FC** | ***p*** |
| Glutamic acid | 31 | 14 | 0,602 | | 0,114 | 1,005 | 0,979 | 1,222 | 0,461 |
| Glycine | 31 | 14 | 1,128 | | 0,172 | 1,080 | 0,468 | 1,102 | 0,515 |
| Alanine | 31 | 14 | 0,843 | | 0,122 | 0,807 | 0,138 | 1,084 | 0,684 |
| Serine | 31 | 14 | 1,059 | | 0,491 | 0,999 | 0,984 | 1,139 | 0,208 |
| Proline | 31 | 14 | 0,548 | | 0,100 | 0,995 | 0,977 | 1,037 | 0,850 |
| Valine | 31 | 14 | 1,166 | | 0,072 | 0,964 | 0,704 | 0,909 | 0,440 |
| Threonine | 31 | 14 | 1,121 | | 0,382 | 1,062 | 0,574 | 0,927 | 0,359 |
| Taurine | 31 | 14 | 1,072 | | 0,622 | 0,801 | 0,171 | 1,167 | 0,380 |
| Isoleucine | 31 | 14 | 1,079 | | 0,526 | 0,937 | 0,579 | 0,953 | 0,780 |
| Leucine | 31 | 14 | 1,155 | | 0,149 | 1,012 | 0,914 | 0,938 | 0,635 |
| Asparagine | 31 | 14 | 1,102 | | 0,336 | 0,932 | 0,417 | 0,905 | 0,399 |
| Aspartic acid | 31 | 14 | 0,478 | | 0,061 | 0,964 | 0,837 | 1,010 | 0,971 |
| Glutamine | 31 | 14 | 1,263 | | 0,097 | 1,090 | 0,397 | 0,827 | 0,168 |
| Lysine | 30 | 14 | 0,979 | | 0,912 | 1,141 | 0,612 | 1,068 | 0,878 |
| Methionine | 31 | 14 | 1,142 | | 0,153 | 1,033 | 0,783 | 0,986 | 0,895 |
| Histidine | 31 | 14 | 0,963 | | 0,771 | 0,976 | 0,827 | 0,862 | 0,214 |
| Phenylalanine | 31 | 14 | 1,095 | | 0,486 | 1,100 | 0,573 | 0,765 | 0,410 |
| Arginine | 31 | 14 | 0,865 | | 0,320 | 0,872 | 0,474 | 1,462 | 0,203 |
| Tyrosine | 31 | 14 | 1,157 | | 0,203 | 0,855 | 0,275 | 0,737 | 0,181 |
| Tryptophan | 31 | 14 | 1,033 | | 0,747 | 0,916 | 0,445 | 0,847 | 0,260 |
| Sarcosine | 31 | 14 | 0,761 | | 0,090 | 0,972 | 0,918 | 1,449 | 0,237 |
| Cystine | 31 | 14 | 0,840 | | 0,176 | 1,062 | 0,622 | 1,035 | 0,865 |
| Gly-DL-Phe | 29 | 14 | 1,035 | | 0,760 | 0,958 | 0,736 | 1,016 | 0,938 |
| Hypotaurine | 30 | 14 | 0,845 | | 0,238 | 0,837 | 0,095 | 1,019 | 0,926 |
| Gamma-Aminobutyric acid | 27 | 10 | 1,462 | | 0,031 | 0,854 | 0,526 | 1,144 | 0,722 |
| DL-2-Aminoadipic acid | 31 | 14 | 1,074 | | 0,658 | 0,900 | 0,575 | 1,154 | 0,557 |
| Kynurenine | 30 | 14 | 0,716 | | 0,030 | 1,225 | 0,220 | 0,722 | 0,384 |
| L-Citrulline | 31 | 14 | 0,810 | | 0,169 | 1,057 | 0,583 | 0,911 | 0,413 |
| L-Homoserine | 31 | 14 | 0,940 | | 0,715 | 1,076 | 0,700 | 0,692 | 0,311 |
| Cys double charge | 31 | 14 | 0,928 | | 0,585 | 1,061 | 0,618 | 1,032 | 0,863 |
| Cystathionine * | 23 | 9 | *0,000* | | *0,000* | *0,000* | *0,000* | *0,000* | *0,000* |
| 1-Methyl-L-histidine | 31 | 14 | 0,839 | | 0,384 | 0,890 | 0,501 | 1,168 | 0,569 |
| D(-)-2-Aminobutyric acid | 31 | 14 | 1,223 | | 0,076 | 0,998 | 0,990 | 1,046 | 0,808 |
| Cer(d18:1/16:0) | 31 | 14 | 1,035 | | 0,905 | 0,466 | 0,031 | 0,757 | 0,599 |
| Cer(d18:1/20:0) | 30 | 13 | 1,495 | | 0,240 | 0,458 | 0,049 | 0,748 | 0,624 |
| Cer(d18:1/21:0) | 30 | 13 | 1,026 | | 0,878 | 0,831 | 0,261 | 1,034 | 0,909 |
| Cer(d18:1/22:0) | 31 | 14 | 1,212 | | 0,497 | 0,483 | 0,040 | 0,778 | 0,639 |
| Cer(d18:1/23:0) | 30 | 13 | 0,641 | | 0,002 | 0,812 | 0,163 | 0,827 | 0,435 |
| Cer(d18:1/24:0) | 31 | 14 | 0,862 | | 0,500 | 0,578 | 0,064 | 0,746 | 0,548 |
| Cer(d18:1/24:1)+Cer(d18:2/24:0) | 28 | 13 | 0,904 | | 0,616 | 0,668 | 0,086 | 0,809 | 0,586 |
| Cer(d18:1/18:0) | 31 | 14 | 1,257 | | 0,233 | 0,641 | 0,062 | 0,795 | 0,598 |
| Cer(d18:1/25:0) * | 18 | 12 | *0,000* | | *0,000* | *0,000* | *0,000* | *0,000* | *0,000* |
| CMH(d18:1/16:0) | 29 | 13 | 1,243 | | 0,604 | 0,441 | 0,055 | 0,748 | 0,656 |
| CMH(d18:1/22:0) | 30 | 14 | 1,824 | | 0,061 | 0,541 | 0,067 | 0,818 | 0,724 |
| CMH(d18:1/24:0) | 30 | 14 | 1,558 | | 0,071 | 0,602 | 0,074 | 0,897 | 0,829 |
| CMH(d18:1/23:0) | 30 | 14 | 1,543 | | 0,008 | 0,817 | 0,225 | 0,996 | 0,986 |
| CMH(d18:1/24:1(2OH)) | 29 | 13 | 1,753 | | 0,008 | 1,109 | 0,661 | 1,102 | 0,739 |
| CMH(d18:1/25:0(2OH)) | 29 | 14 | 0,857 | | 0,413 | 0,986 | 0,939 | 1,088 | 0,759 |
| ChoE(18:1) * | 0 | 7 | *0,000* | | *0,000* | *0,000* | *0,000* | *0,000* | *0,000* |
| ChoE(18:2) * | 9 | 8 | *0,000* | | *0,000* | *0,000* | *0,000* | *0,000* | *0,000* |
| ChoE(18:3) * | 0 | 5 | *0,000* | | *0,000* | *0,000* | *0,000* | *0,000* | *0,000* |
| ChoE(20:4) * | 0 | 7 | *0,000* | | *0,000* | *0,000* | *0,000* | *0,000* | *0,000* |
| ChoE(20:5) * | 0 | 7 | *0,000* | | *0,000* | *0,000* | *0,000* | *0,000* | *0,000* |
| ChoE(22:6) * | 3 | 7 | *0,000* | | *0,000* | *0,000* | *0,000* | *0,000* | *0,000* |
| PC(16:0/16:0) | 31 | 14 | 0,544 | | 0,046 | 0,796 | 0,169 | 0,832 | 0,471 |
| PC(32:1) | 31 | 14 | 0,402 | | 0,009 | 0,805 | 0,204 | 1,001 | 0,996 |
| PC(14:0/18:2) | 27 | 13 | 0,174 | | 0,003 | 1,057 | 0,771 | 0,860 | 0,545 |
| PC(16:0/18:0) | 31 | 14 | 0,400 | | 0,027 | 0,759 | 0,086 | 0,784 | 0,366 |
| PC(16:0/18:1) | 31 | 14 | 0,705 | | 0,039 | 0,808 | 0,140 | 0,872 | 0,512 |
| PC(16:0/18:2) | 31 | 14 | 0,279 | | 0,006 | 0,878 | 0,401 | 0,879 | 0,509 |
| PC(14:0/20:4) | 9 | 11 | 0,237 | | 0,021 | 1,013 | 0,975 | 0,000 | 0,000 |
| PC(18:0/18:1) | 31 | 14 | 0,525 | | 0,019 | 0,800 | 0,134 | 0,880 | 0,534 |
| PC(18:0/18:2) | 31 | 14 | 0,210 | | 0,008 | 0,892 | 0,472 | 0,860 | 0,390 |
| PC(18:2/18:2) | 30 | 14 | 0,195 | | 0,012 | 1,118 | 0,613 | 0,861 | 0,580 |
| PC(16:0/20:4) | 31 | 14 | 0,215 | | 0,017 | 1,048 | 0,814 | 0,976 | 0,938 |
| PC(16:0/20:5) | 30 | 14 | 0,191 | | 0,011 | 0,711 | 0,164 | 1,160 | 0,656 |
| PC(18:0/20:3) | 31 | 14 | 0,110 | | 0,009 | 0,960 | 0,835 | 0,728 | 0,167 |
| PC(18:0/20:4) | 31 | 14 | 0,197 | | 0,015 | 1,146 | 0,409 | 0,948 | 0,816 |
| PC(38:5) 1 | 31 | 14 | 0,266 | | 0,018 | 1,003 | 0,985 | 1,079 | 0,788 |
| PC(38:5) 2 | 28 | 14 | 0,128 | | 0,012 | 0,879 | 0,548 | 1,173 | 0,630 |
| PC(18:2/20:4) | 30 | 14 | 0,192 | | 0,013 | 1,143 | 0,520 | 1,403 | 0,240 |
| PC(16:0/22:6) | 31 | 14 | 0,085 | | 0,012 | 0,890 | 0,520 | 0,806 | 0,441 |
| PC(18:0/22:4) | 27 | 12 | 0,123 | | 0,011 | 0,940 | 0,747 | 0,851 | 0,460 |
| PC(20:0/20:4) * | 18 | 10 | *0,000* | | *0,000* | *0,000* | *0,000* | *0,000* | *0,000* |
| PC(40:5) * | 15 | 10 | *0,000* | | *0,000* | *0,000* | *0,000* | *0,000* | *0,000* |
| PC(18:0/22:5) | 30 | 14 | 0,230 | | 0,014 | 1,037 | 0,816 | 0,912 | 0,667 |
| PC(18:0/22:6) | 31 | 14 | 0,068 | | 0,015 | 1,144 | 0,533 | 0,756 | 0,097 |
| PC(18:1/22:6) | 26 | 12 | 0,117 | | 0,009 | 1,045 | 0,801 | 0,750 | 0,236 |
| PC(40:8) * | 11 | 11 | *0,000* | | *0,000* | *0,000* | *0,000* | *0,000* | *0,000* |
| PC(16:1/18:2) | 26 | 12 | 0,197 | | 0,015 | 0,964 | 0,870 | 1,070 | 0,799 |
| PC(34:3) | 30 | 14 | 0,455 | | 0,020 | 0,889 | 0,577 | 1,029 | 0,925 |
| PC(36:3) | 31 | 14 | 0,271 | | 0,005 | 0,881 | 0,470 | 0,904 | 0,626 |
| PC(31:0) | 29 | 13 | 0,518 | | 0,048 | 0,787 | 0,121 | 0,753 | 0,251 |
| PC(30:0) | 31 | 14 | 0,524 | | 0,005 | 0,649 | 0,028 | 0,656 | 0,171 |
| PE(16:0/18:1) | 31 | 14 | 1,269 | | 0,231 | 0,790 | 0,294 | 0,858 | 0,691 |
| PE(16:0/18:2) | 31 | 14 | 0,730 | | 0,305 | 0,766 | 0,231 | 0,846 | 0,663 |
| PC(16:0/17:0) | 28 | 11 | 0,713 | | 0,210 | 0,624 | 0,066 | 0,793 | 0,543 |
| PC(33:1) | 30 | 14 | 0,525 | | 0,014 | 0,776 | 0,094 | 0,888 | 0,558 |
| PE(18:1/18:2) | 31 | 14 | 1,037 | | 0,875 | 0,833 | 0,424 | 0,959 | 0,901 |
| PE(16:0/20:4) | 31 | 14 | 1,006 | | 0,977 | 0,869 | 0,582 | 0,849 | 0,723 |
| PE(18:2/18:2) | 30 | 12 | 0,922 | | 0,764 | 0,930 | 0,819 | 0,836 | 0,736 |
| PE(20:5/16:0) | 30 | 14 | 1,379 | | 0,354 | 0,641 | 0,222 | 0,945 | 0,930 |
| PC(17:0/18:1) | 24 | 10 | 0,711 | | 0,207 | 0,983 | 0,933 | 1,059 | 0,871 |
| PC(16:0/19:1) | 30 | 14 | 0,597 | | 0,026 | 0,952 | 0,750 | 0,984 | 0,930 |
| PC(17:1/18:1) * | 21 | 12 | *0,000* | | *0,000* | *0,000* | *0,000* | *0,000* | *0,000* |
| PC(17:0/18:2) | 29 | 13 | 0,341 | | 0,003 | 1,006 | 0,974 | 1,044 | 0,856 |
| PE(18:0/20:4) | 31 | 14 | 1,140 | | 0,483 | 0,924 | 0,719 | 0,921 | 0,815 |
| PE(20:4/18:2) | 31 | 14 | 1,275 | | 0,446 | 0,701 | 0,223 | 1,198 | 0,688 |
| PE(16:0/22:6) | 31 | 14 | 0,880 | | 0,535 | 0,832 | 0,442 | 0,805 | 0,604 |
| PC(37:2) | 31 | 14 | 0,717 | | 0,082 | 1,097 | 0,610 | 0,991 | 0,973 |
| PC(17:0/20:4) | 26 | 13 | 0,347 | | 0,021 | 1,357 | 0,170 | 1,063 | 0,881 |
| PE(18:0/22:6) | 31 | 13 | 0,651 | | 0,154 | 1,101 | 0,734 | 0,868 | 0,728 |
| PE(18:0/18:1) | 31 | 14 | 0,988 | | 0,949 | 0,792 | 0,180 | 0,903 | 0,672 |
| PI(18:0/20:3) | 26 | 11 | 0,485 | | 0,046 | 1,004 | 0,994 | 1,085 | 0,880 |
| PI(18:0/20:4) | 30 | 14 | 0,671 | | 0,194 | 1,156 | 0,694 | 1,452 | 0,387 |
| PI(18:0/22:6) | 23 | 11 | 0,743 | | 0,172 | 0,679 | 0,235 | 1,092 | 0,868 |
| DG(34:1) | 31 | 14 | 1,223 | | 0,286 | 1,288 | 0,368 | 0,738 | 0,396 |
| DG(36:3) | 24 | 13 | 1,481 | | 0,375 | 2,446 | 0,208 | 0,907 | 0,879 |
| Octadecanamide | 31 | 14 | 0,506 | | 0,121 | 2,606 | 0,074 | 0,693 | 0,567 |
| 20:2n-x amide | 28 | 13 | 0,894 | | 0,643 | 1,211 | 0,530 | 0,469 | 0,124 |
| 23:1n-x amide | 31 | 14 | 1,151 | | 0,524 | 1,359 | 0,359 | 0,666 | 0,468 |
| 24:1n-x amide | 31 | 14 | 1,375 | | 0,163 | 1,221 | 0,529 | 0,603 | 0,361 |
| PC(O-16:0/14:0) * | 11 | 7 | *0,000* | | *0,000* | *0,000* | *0,000* | *0,000* | *0,000* |
| PC(O-16:0/16:0) | 31 | 14 | 0,087 | | 0,032 | 0,904 | 0,568 | 0,853 | 0,507 |
| PC(P-16:0/16:0) | 29 | 12 | 0,417 | | 0,005 | 0,891 | 0,464 | 0,866 | 0,559 |
| PC(O-34:0) | 27 | 12 | 0,088 | | 0,043 | 0,884 | 0,553 | 0,741 | 0,353 |
| PC(O-34:1) | 31 | 14 | 0,294 | | 0,028 | 0,812 | 0,194 | 0,983 | 0,935 |
| PC(O-16:0/18:2) | 26 | 12 | 0,078 | | 0,009 | 1,018 | 0,933 | 0,916 | 0,653 |
| PC(P-16:0/18:2) | 30 | 14 | 0,309 | | 0,013 | 1,024 | 0,885 | 1,034 | 0,867 |
| PC(O-18:1/18:1) | 29 | 12 | 0,192 | | 0,024 | 0,788 | 0,234 | 0,793 | 0,274 |
| PC(O-18:0/18:2) * | 15 | 9 | *0,000* | | *0,000* | *0,000* | *0,000* | *0,000* | *0,000* |
| PC(O-18:1/18:2) * | 21 | 10 | *0,000* | | *0,000* | *0,000* | *0,000* | *0,000* | *0,000* |
| PC(O-16:0/20:4) | 30 | 13 | 0,096 | | 0,006 | 0,796 | 0,266 | 0,948 | 0,847 |
| PC(P-16:0/20:4) | 29 | 14 | 0,384 | | 0,018 | 1,145 | 0,520 | 1,075 | 0,827 |
| PC(O-18:0/20:4) | 28 | 12 | 0,082 | | 0,003 | 0,893 | 0,568 | 0,932 | 0,814 |
| PC(O-38:5) | 29 | 14 | 0,142 | | 0,008 | 0,984 | 0,901 | 0,937 | 0,692 |
| PC(P-18:0/20:4) | 29 | 13 | 0,262 | | 0,015 | 1,007 | 0,974 | 0,909 | 0,724 |
| PC(O-22:1/20:4) * | 5 | 7 | *0,000* | | *0,000* | *0,000* | *0,000* | *0,000* | *0,000* |
| PC(O-24:1/20:4) * | 19 | 11 | *0,000* | | *0,000* | *0,000* | *0,000* | *0,000* | *0,000* |
| PE(O-16:0/18:1) | 31 | 14 | 1,373 | | 0,103 | 0,896 | 0,624 | 0,960 | 0,906 |
| PE(P-16:0/18:2) | 31 | 14 | 1,038 | | 0,867 | 0,773 | 0,334 | 1,013 | 0,974 |
| PE(P-18:0/18:1) | 31 | 14 | 1,169 | | 0,351 | 0,871 | 0,407 | 0,962 | 0,861 |
| PE(P-16:0/20:4) | 31 | 14 | 1,387 | | 0,091 | 1,030 | 0,904 | 0,971 | 0,936 |
| PE(P-18:0/20:4) | 31 | 14 | 1,405 | | 0,054 | 0,995 | 0,981 | 1,011 | 0,971 |
| PE(P-18:1/20:4) | 31 | 14 | 1,364 | | 0,094 | 0,999 | 0,996 | 1,044 | 0,899 |
| PE(P-16:0/22:6) | 31 | 14 | 0,862 | | 0,476 | 0,730 | 0,212 | 0,684 | 0,450 |
| PE(18:1e/22:4) | 25 | 13 | 0,648 | | 0,163 | 0,835 | 0,456 | 0,931 | 0,840 |
| PE(P-20:0/20:4) | 30 | 14 | 1,157 | | 0,402 | 0,957 | 0,804 | 1,023 | 0,930 |
| PE(P-18:0/22:5) + PE(P-20:1/20:4) | 29 | 13 | 0,812 | | 0,367 | 0,924 | 0,656 | 1,023 | 0,944 |
| PE(P-18:0/18:2) | 31 | 14 | 0,913 | | 0,698 | 0,740 | 0,202 | 1,037 | 0,915 |
| PE(18:1e/22:6) | 31 | 14 | 0,781 | | 0,246 | 0,719 | 0,170 | 0,717 | 0,490 |
| SM(d18:0/14:0) | 30 | 14 | 1,044 | | 0,754 | 0,766 | 0,086 | 0,885 | 0,559 |
| SM(d18:0/15:0) | 22 | 12 | 0,697 | | 0,082 | 1,107 | 0,533 | 0,997 | 0,988 |
| SM(d18:0/16:0) | 31 | 14 | 0,706 | | 0,087 | 0,781 | 0,228 | 0,997 | 0,992 |
| SM(d18:0/18:0) | 30 | 14 | 0,875 | | 0,529 | 0,801 | 0,276 | 0,893 | 0,727 |
| SM(d18:0/22:0) | 31 | 14 | 1,372 | | 0,066 | 0,814 | 0,384 | 0,972 | 0,931 |
| SM(d18:1/12:0) | 31 | 14 | 0,714 | | 0,053 | 0,744 | 0,079 | 0,746 | 0,272 |
| SM(32:1) | 31 | 14 | 1,003 | | 0,984 | 0,727 | 0,053 | 0,789 | 0,359 |
| SM(33:1) | 31 | 14 | 0,950 | | 0,728 | 0,812 | 0,151 | 0,878 | 0,473 |
| SM(d18:1/16:0) | 31 | 14 | 1,058 | | 0,668 | 0,792 | 0,117 | 0,941 | 0,764 |
| SM(d18:2/16:0) | 31 | 14 | 0,715 | | 0,055 | 0,867 | 0,381 | 0,917 | 0,731 |
| SM(d18:1/17:0) | 31 | 14 | 0,947 | | 0,716 | 0,775 | 0,123 | 0,895 | 0,626 |
| SM(d18:1/18:0) | 31 | 14 | 1,090 | | 0,590 | 0,765 | 0,113 | 0,861 | 0,551 |
| SM(36:2) | 31 | 14 | 0,635 | | 0,037 | 0,854 | 0,439 | 0,856 | 0,636 |
| SM(38:1) | 31 | 14 | 1,495 | | 0,009 | 0,767 | 0,096 | 0,955 | 0,843 |
| SM(d18:2/20:0) | 31 | 14 | 1,033 | | 0,835 | 0,828 | 0,275 | 0,942 | 0,802 |
| SM(39:1) | 31 | 14 | 1,512 | | 0,007 | 0,861 | 0,400 | 1,015 | 0,947 |
| SM(d18:1/22:0) | 31 | 14 | 1,483 | | 0,003 | 0,844 | 0,235 | 1,045 | 0,781 |
| SM(d16:1/24:1) | 31 | 14 | 0,833 | | 0,458 | 0,745 | 0,256 | 0,761 | 0,484 |
| SM(d18:2/22:0) | 31 | 14 | 0,988 | | 0,938 | 0,845 | 0,339 | 0,938 | 0,786 |
| SM(d18:1/23:0) | 31 | 14 | 1,541 | | 0,007 | 0,937 | 0,732 | 1,096 | 0,701 |
| SM(d18:1/23:1) | 31 | 14 | 0,948 | | 0,771 | 0,844 | 0,338 | 0,905 | 0,586 |
| SM(d18:2/23:0) | 31 | 14 | 1,006 | | 0,975 | 0,801 | 0,341 | 1,274 | 0,451 |
| SM(d18:1/24:1) + SM(d18:2/24:0) | 31 | 14 | 1,010 | | 0,941 | 0,865 | 0,346 | 1,022 | 0,897 |
| SM(42:3) | 31 | 14 | 0,756 | | 0,103 | 0,911 | 0,609 | 0,963 | 0,861 |
| SM(d18:2/14:0) | 26 | 12 | 0,421 | | 0,003 | 0,955 | 0,760 | 0,936 | 0,792 |
| SM(42:1) | 31 | 14 | 1,388 | | 0,016 | 0,847 | 0,283 | 1,023 | 0,909 |
| SM(d18:1/25:0) | 31 | 14 | 1,333 | | 0,055 | 0,876 | 0,472 | 1,013 | 0,959 |
| SM(43:1) | 29 | 14 | 1,189 | | 0,347 | 1,023 | 0,891 | 0,815 | 0,259 |
| SM(43:2) | 30 | 14 | 0,880 | | 0,558 | 1,003 | 0,986 | 0,838 | 0,450 |
| SM(38:0) | 31 | 14 | 1,301 | | 0,124 | 0,715 | 0,135 | 0,945 | 0,864 |
| SM(31:1) | 29 | 13 | 1,013 | | 0,939 | 0,843 | 0,287 | 0,809 | 0,223 |
| TG(50:4) * | 12 | 8 | *0,000* | | *0,000* | *0,000* | *0,000* | *0,000* | *0,000* |
| TG(53:3) * | 16 | 12 | *0,000* | | *0,000* | *0,000* | *0,000* | *0,000* | *0,000* |
| TG(56:3) | 27 | 13 | 0,606 | | 0,200 | 1,219 | 0,464 | 0,935 | 0,857 |
| TG(56:5) * | 0 | 6 | *0,000* | | *0,000* | *0,000* | *0,000* | *0,000* | *0,000* |
| TG(56:6) * | 0 | 6 | *0,000* | | *0,000* | *0,000* | *0,000* | *0,000* | *0,000* |
| TG(56:7) 1 * | 0 | 7 | *0,000* | | *0,000* | *0,000* | *0,000* | *0,000* | *0,000* |
| TG(56:7) 2 * | 1 | 7 | *0,000* | | *0,000* | *0,000* | *0,000* | *0,000* | *0,000* |
| TG(56:8) | 7 | 8 | 0,084 | | 0,107 | 1,005 | 0,990 | 0,000 | 0,000 |
| TG(58:9) * | 0 | 7 | *0,000* | | *0,000* | *0,000* | *0,000* | *0,000* | *0,000* |
| L-Octanoylcarnitine | 28 | 14 | 0,948 | | 0,839 | 1,599 | 0,093 | 1,039 | 0,938 |
| Decanoylcarnitine | 28 | 12 | 1,478 | | 0,165 | 1,314 | 0,398 | 1,900 | 0,281 |
| Dodecanoylcarnitine | 30 | 14 | 2,246 | | 0,006 | 1,472 | 0,252 | 0,901 | 0,881 |
| AC(14:1n-x) | 26 | 11 | 2,344 | | 0,008 | 1,710 | 0,157 | 1,011 | 0,988 |
| AC(8:1n-x) | 31 | 14 | 1,142 | | 0,694 | 1,916 | 0,155 | 0,489 | 0,516 |
| AC(12:1n-x) | 30 | 14 | 1,707 | | 0,018 | 1,245 | 0,391 | 1,486 | 0,263 |
| AC(14:2n-x) | 29 | 13 | 2,154 | | 0,017 | 1,762 | 0,146 | 1,264 | 0,642 |
| Stearoylcarnitine | 31 | 14 | 0,838 | | 0,670 | 0,393 | 0,052 | 1,664 | 0,312 |
| Cholic acid (31% bile) | 31 | 14 | 1,256 | | 0,600 | 0,897 | 0,847 | 0,421 | 0,175 |
| Deoxycholic Acid | 31 | 14 | 0,753 | | 0,250 | 0,662 | 0,213 | 0,602 | 0,238 |
| Chenodeoxycholic acid | 26 | 13 | 0,556 | | 0,201 | 0,896 | 0,841 | 0,497 | 0,257 |
| UDCA + HDCA * | 16 | 11 | *0,000* | | *0,000* | *0,000* | *0,000* | *0,000* | *0,000* |
| Litocholic acid | 30 | 13 | 1,019 | | 0,923 | 1,004 | 0,983 | 0,945 | 0,782 |
| Taurocholic qcid | 25 | 11 | 0,782 | | 0,538 | 0,793 | 0,577 | 0,472 | 0,299 |
| Taurodeoxycholic acid | 28 | 12 | 1,125 | | 0,729 | 0,813 | 0,514 | 1,448 | 0,382 |
| TLCA * | 14 | 9 | *0,000* | | *0,000* | *0,000* | *0,000* | *0,000* | *0,000* |
| Glycocholic acid | 31 | 14 | 1,626 | | 0,136 | 1,702 | 0,170 | 0,758 | 0,179 |
| Glycochenodeoxycholic acid | 30 | 13 | 0,832 | | 0,498 | 0,852 | 0,469 | 0,702 | 0,127 |
| Glycodeoxycholic acid | 29 | 14 | 1,260 | | 0,438 | 0,966 | 0,918 | 1,024 | 0,956 |
| Arachidonic acid | 31 | 14 | 0,333 | | 0,042 | 1,084 | 0,568 | 1,079 | 0,732 |
| PUFA 16:3n-x | 31 | 14 | 2,062 | | 0,012 | 0,628 | 0,124 | 1,475 | 0,356 |
| 9,10-DiHOME * | 15 | 10 | *0,000* | | *0,000* | *0,000* | *0,000* | *0,000* | *0,000* |
| PC(15:0/0:0) * | 12 | 10 | *0,000* | | *0,000* | *0,000* | *0,000* | *0,000* | *0,000* |
| PC(16:0/0:0) | 31 | 14 | 0,401 | | 0,057 | 0,563 | 0,121 | 0,736 | 0,607 |
| PC(0:0/16:0) | 31 | 14 | 0,346 | | 0,065 | 0,648 | 0,181 | 0,806 | 0,682 |
| PC(0:0/18:0) | 31 | 14 | 0,338 | | 0,033 | 0,694 | 0,212 | 0,953 | 0,919 |
| PC(18:0/0:0) | 31 | 14 | 0,251 | | 0,022 | 0,680 | 0,188 | 0,801 | 0,664 |
| PC(0:0/18:1) | 31 | 14 | 0,226 | | 0,081 | 1,069 | 0,784 | 0,922 | 0,845 |
| PC(18:1/0:0) | 31 | 14 | 0,320 | | 0,050 | 0,760 | 0,240 | 0,776 | 0,512 |
| PC(0:0/18:2) | 31 | 14 | 0,157 | | 0,093 | 1,106 | 0,711 | 1,063 | 0,880 |
| PC(18:2/0:0) | 25 | 11 | 0,992 | | 0,965 | 0,872 | 0,436 | 1,100 | 0,694 |
| PC(20:0/0:0) | 27 | 13 | 0,428 | | 0,049 | 0,812 | 0,553 | 0,526 | 0,402 |
| PC(20:1/0:0) | 28 | 13 | 0,834 | | 0,600 | 0,553 | 0,174 | 0,804 | 0,735 |
| PC(20:2/0:0) * | 13 | 9 | *0,000* | | *0,000* | *0,000* | *0,000* | *0,000* | *0,000* |
| PC(0:0/20:3) | 8 | 10 | 0,096 | | 0,112 | 2,063 | 0,352 | 0,000 | 0,000 |
| PC(20:3/0:0) * | 2 | 6 | *0,000* | | *0,000* | *0,000* | *0,000* | *0,000* | *0,000* |
| PC(0:0/20:4) | 30 | 14 | 0,146 | | 0,055 | 1,440 | 0,123 | 0,829 | 0,681 |
| PC(20:4/0:0) | 22 | 13 | 0,162 | | 0,113 | 1,951 | 0,131 | 1,238 | 0,596 |
| PC(0:0/22:6) * | 5 | 8 | *0,000* | | *0,000* | *0,000* | *0,000* | *0,000* | *0,000* |
| PC(22:6/0:0) * | 3 | 6 | *0,000* | | *0,000* | *0,000* | *0,000* | *0,000* | *0,000* |
| PC(17:0/0:0) | 31 | 13 | 0,370 | | 0,009 | 0,712 | 0,169 | 0,928 | 0,846 |
| PC(0:0/17:0) * | 11 | 8 | *0,000* | | *0,000* | *0,000* | *0,000* | *0,000* | *0,000* |
| LPC(22:0) * | 6 | 7 | *0,000* | | *0,000* | *0,000* | *0,000* | *0,000* | *0,000* |
| PE(14:0/0:0) * | 9 | 8 | *0,000* | | *0,000* | *0,000* | *0,000* | *0,000* | *0,000* |
| PE(0:0/16:0) | 31 | 14 | 0,852 | | 0,492 | 0,689 | 0,221 | 0,805 | 0,649 |
| PE(16:0/0:0) | 31 | 14 | 0,987 | | 0,965 | 0,576 | 0,163 | 0,701 | 0,576 |
| PE(0:0/16:1) | 31 | 14 | 0,535 | | 0,200 | 0,850 | 0,715 | 1,458 | 0,515 |
| PE(0:0/18:0) | 31 | 14 | 1,043 | | 0,897 | 0,474 | 0,117 | 0,805 | 0,735 |
| PE(18:0/0:0) | 31 | 14 | 1,004 | | 0,987 | 0,579 | 0,153 | 0,712 | 0,579 |
| PE(0:0/18:1) | 31 | 14 | 1,035 | | 0,864 | 0,982 | 0,949 | 0,560 | 0,374 |
| PE(18:1/0:0) | 31 | 14 | 0,695 | | 0,221 | 0,710 | 0,289 | 0,841 | 0,738 |
| PE(0:0/18:2) | 31 | 14 | 0,718 | | 0,224 | 0,890 | 0,699 | 0,632 | 0,443 |
| PE(18:2/0:0) | 28 | 12 | 0,340 | | 0,149 | 0,633 | 0,196 | 1,407 | 0,514 |
| PE(0:0/20:3) | 31 | 14 | 0,693 | | 0,078 | 0,953 | 0,820 | 0,588 | 0,242 |
| PE(20:3/0:0) * | 10 | 8 | *0,000* | | *0,000* | *0,000* | *0,000* | *0,000* | *0,000* |
| PE(0:0/20:4) | 31 | 14 | 1,213 | | 0,380 | 1,073 | 0,832 | 0,556 | 0,414 |
| PE(20:4/0:0) | 31 | 14 | 0,350 | | 0,105 | 0,769 | 0,542 | 1,693 | 0,431 |
| LPE(20:5) | 28 | 13 | 1,276 | | 0,367 | 0,914 | 0,817 | 0,539 | 0,507 |
| PE(0:0/22:4) | 31 | 14 | 0,665 | | 0,178 | 1,057 | 0,838 | 0,613 | 0,386 |
| PE(22:5/0:0) | 31 | 14 | 0,708 | | 0,146 | 0,968 | 0,878 | 0,727 | 0,538 |
| PE(22:5/0:0) * | 14 | 6 | *0,000* | | *0,000* | *0,000* | *0,000* | *0,000* | *0,000* |
| PE(0:0/22:6) | 31 | 14 | 0,775 | | 0,249 | 0,860 | 0,650 | 0,497 | 0,365 |
| PE(22:6/0:0) | 27 | 13 | 0,319 | | 0,106 | 0,724 | 0,427 | 1,364 | 0,577 |
| LPI(16:0) | 24 | 11 | 0,691 | | 0,232 | 0,701 | 0,305 | 0,752 | 0,646 |
| LPI(18:0) | 31 | 14 | 0,631 | | 0,059 | 0,665 | 0,136 | 1,198 | 0,672 |
| LPI(20:4) * | 9 | 6 | *0,000* | | *0,000* | *0,000* | *0,000* | *0,000* | *0,000* |
| PC(O-16:0/0:0) | 31 | 13 | 0,139 | | 0,045 | 1,152 | 0,357 | 1,126 | 0,639 |
| PC(P-16:0/0:0) * | 16 | 11 | *0,000* | | *0,000* | *0,000* | *0,000* | *0,000* | *0,000* |
| PC(O-18:0/0:0) * | 21 | 12 | *0,000* | | *0,000* | *0,000* | *0,000* | *0,000* | *0,000* |
| PC(O-18:1/0:0) * | 18 | 11 | *0,000* | | *0,000* | *0,000* | *0,000* | *0,000* | *0,000* |
| PC(P-18:0/0:0) * | 5 | 8 | *0,000* | | *0,000* | *0,000* | *0,000* | *0,000* | *0,000* |
| PC(O-20:0/0:0) * | 5 | 8 | *0,000* | | *0,000* | *0,000* | *0,000* | *0,000* | *0,000* |
| PC(O-20:1/0:0) * | 2 | 6 | *0,000* | | *0,000* | *0,000* | *0,000* | *0,000* | *0,000* |
| PC(O-22:1/0:0) * | 2 | 7 | *0,000* | | *0,000* | *0,000* | *0,000* | *0,000* | *0,000* |
| PC(O-24:1/0:0) * | 15 | 10 | *0,000* | | *0,000* | *0,000* | *0,000* | *0,000* | *0,000* |
| PC(O-24:2/0:0) * | 4 | 7 | *0,000* | | *0,000* | *0,000* | *0,000* | *0,000* | *0,000* |
| PE(O-16:0/0:0) | 27 | 14 | 0,600 | | 0,127 | 0,704 | 0,241 | 0,905 | 0,835 |
| PE(P-16:1/0:0) | 31 | 14 | 0,590 | | 0,063 | 0,788 | 0,374 | 0,841 | 0,699 |
| PE(P-18:0/0:0) | 31 | 14 | 0,414 | | 0,040 | 0,691 | 0,157 | 0,818 | 0,660 |
| PE(P-20:0/0:0) * | 16 | 12 | *0,000* | | *0,000* | *0,000* | *0,000* | *0,000* | *0,000* |
| PE(P-20:1/0:0) * | 9 | 7 | *0,000* | | *0,000* | *0,000* | *0,000* | *0,000* | *0,000* |
| Cortisol (hydrocortisone) | 31 | 14 | 1,286 | | 0,251 | 0,806 | 0,403 | 1,351 | 0,405 |
| DHEAS | 31 | 14 | 3,774 | | 0,012 | 0,547 | 0,261 | 0,885 | 0,876 |
| Pregnenolone sulfate | 30 | 14 | 1,583 | | 0,297 | 1,256 | 0,616 | 0,822 | 0,706 |
| isomer androsterone sulfate 1 | 31 | 13 | 3,272 | | 0,019 | 0,639 | 0,412 | 1,006 | 0,994 |
| isomer androsterone sulfate 2 | 31 | 14 | 2,812 | | 0,000 | 0,964 | 0,865 | 1,317 | 0,334 |
| androsterone sulfate + etiocholanolone sulfate | 31 | 14 | 2,217 | | 0,001 | 1,006 | 0,978 | 1,859 | 0,023 |
| isomer androsterone sulfate 3 | 31 | 14 | 1,220 | | 0,474 | 0,833 | 0,554 | 1,055 | 0,927 |
| isomer pregn-5-ene-3,20-diol sulphate | 26 | 12 | 3,202 | | 0,120 | 0,289 | 0,204 | 0,545 | 0,617 |
| Phenylacetic acid | 31 | 13 | 0,632 | | 0,097 | 1,194 | 0,359 | 0,638 | 0,305 |
| N-Acetylneuraminic acid | 31 | 14 | 0,809 | | 0,074 | 0,893 | 0,256 | 1,236 | 0,140 |
| Suberic acid | 31 | 14 | 0,599 | | 0,045 | 1,268 | 0,451 | 0,605 | 0,250 |
| 3-Hydroxyanthranilic acid | 28 | 13 | 0,602 | | 0,234 | 0,668 | 0,323 | 1,718 | 0,372 |
| 4-Pyridoxic acid | 31 | 14 | 0,842 | | 0,245 | 0,863 | 0,489 | 1,089 | 0,788 |
| 3-Hydroxyglutaric acid | 30 | 13 | 0,987 | | 0,920 | 1,113 | 0,568 | 0,828 | 0,291 |
| 2-PG, 3-PG | 30 | 14 | 0,803 | | 0,154 | 1,031 | 0,884 | 0,636 | 0,103 |
| ADP | 31 | 13 | 1,175 | | 0,266 | 0,947 | 0,701 | 0,831 | 0,360 |
| AMP | 31 | 14 | 0,730 | | 0,171 | 1,029 | 0,894 | 0,782 | 0,368 |
| 6-Phosphogluconate | 30 | 14 | 0,992 | | 0,935 | 1,132 | 0,225 | 1,182 | 0,157 |
| Succinate | 31 | 14 | 1,211 | | 0,110 | 1,051 | 0,719 | 1,019 | 0,932 |
| Citrate/iso-Citrate | 31 | 14 | 1,301 | | 0,035 | 0,931 | 0,470 | 1,238 | 0,112 |
| Malate | 29 | 12 | 0,922 | | 0,594 | 0,938 | 0,763 | 1,060 | 0,861 |
| Alpha-Ketoglutarate | 30 | 13 | 1,105 | | 0,643 | 1,130 | 0,526 | 1,035 | 0,829 |
| Fumaric acid | 29 | 14 | 1,000 | | 0,998 | 0,951 | 0,763 | 1,501 | 0,081 |
| Pyruvate | 31 | 14 | 1,089 | | 0,643 | 0,989 | 0,946 | 1,163 | 0,443 |
| NAD * | 20 | 12 | *0,000* | | *0,000* | *0,000* | *0,000* | *0,000* | *0,000* |
| Caffeic acid | 26 | 12 | 1,817 | | 0,084 | 1,408 | 0,508 | 0,649 | 0,316 |
| Ascorbic acid * | 4 | 5 | *0,000* | | *0,000* | *0,000* | *0,000* | *0,000* | *0,000* |
| Hydroxyphenyllactic acid | 31 | 14 | 0,978 | | 0,919 | 1,394 | 0,353 | 0,968 | 0,900 |
| Sorbitol | 31 | 14 | 0,667 | | 0,161 | 0,872 | 0,708 | 0,614 | 0,462 |
| Cytidine | 28 | 12 | 0,803 | | 0,256 | 1,291 | 0,301 | 0,897 | 0,746 |
| Inosine | 31 | 14 | 0,922 | | 0,692 | 0,896 | 0,580 | 1,187 | 0,541 |
| Xanthosine | 31 | 14 | 1,653 | | 0,003 | 0,856 | 0,442 | 0,691 | 0,095 |
| UDP-Glucose | 22 | 11 | 0,605 | | 0,064 | 0,981 | 0,944 | 1,221 | 0,650 |
| cAMP | 31 | 14 | 1,784 | | 0,010 | 0,867 | 0,585 | 0,551 | 0,045 |
| Uracil | 31 | 14 | 1,256 | | 0,070 | 0,885 | 0,397 | 1,037 | 0,836 |
| Uridine | 30 | 13 | 0,986 | | 0,920 | 0,966 | 0,829 | 0,884 | 0,679 |
| Riboflavin * | 27 | 8 | *0,000* | | *0,000* | *0,000* | *0,000* | *0,000* | *0,000* |
| Pantothenic acid | 31 | 14 | 1,122 | | 0,336 | 1,123 | 0,332 | 1,120 | 0,517 |
