# Supplementary material for: Metabolic alterations in urine extracellular vesicles are associated to prostate cancer pathogenesis and progression
Source: J Extracell Vesicles. 2018 May 7;7(1):1470442. doi: 10.1080/20013078.2018.1470442 (PMC5944373; doi:10.1080/20013078.2018.1470442)
Supplement: Supplemental_files.zip [file ZJEV_A_1470442_SM6791.zip › Supplemental files/SUPPLEMENTARY_TABLE_2.docx]

**Supplementary Table 2.** Enzymes that were identified for each one of the metabolites that showed significant differences between PCa and BPH and the genes related to them.

| Metabolite | Enzymes (EC code) | Genes |
| --- | --- | --- |
| Gamma-Aminobutyric acid | EC 1.2.1.3 | ALDH3A2; ALDH2; ALDH9A1; ALDH7A1; ALDH1B1 |
|  | EC 2.1.4.1 | GATM |
|  | EC 2.6.1.19 | ABAT |
|  | EC 3.4.13.18 | CNDP2 |
|  | EC 3.4.13.20 | CNDP1 |
|  | EC 4.1.1.15 | GAD1; GAD2 |
|  | EC 6.3.2.11 | CARNS1 |
| Kynurenine | EC 1.14.13.9 | KMO |
|  | EC 2.6.1.7 | AADAT; KYAT3; KYAT1 |
|  | EC 3.5.1.9 | AFMID |
|  | EC 3.7.1.3 | KYNU |
| Citrate / iso-Citrate | EC 2.3.3.1 | CS |
|  | EC 2.3.3.8 | ACLY |
|  | EC 4.2.1.3 | ACO2; ACO1 |
|  | EC 6.3.1.17 | RIMKLB |
| PC(16:0/16:0), PC(14:0/18:2), PC(16:0/18:0), PC(16:0/18:1), PC(16:0/18:2), PC(14:0/20:4), PC(18:0/18:1), PC(18:0/18:2), PC(18:2/18:2), PC(16:0/20:4), PC(16:0/20:5), PC(18:0/20:3), PC(18:0/20:4), PC(18:2/20:4), PC(16:0/22:6), PC(18:0/22:4), PC(18:0/22:5), PC(18:0/22:6), PC(18:1/22:6), PC(16:1/18:2) | EC 2.3.1.43  EC 2.3.1.135  EC 2.7.8.2  EC 2.7.8.27  EC 3.1.1.32  EC 3.1.4.4 | LCAT  LRAT  CEPT1; CHPT1  SGMS2; SGMS1  PLA2G16  PLD2; PLD4; PLD1; PLD3 |
| Arachidonic acid | EC 1.13.11.31 | ALOX12 |
|  | EC 1.13.11.33 | ALOX15; ALOX15B |
|  | EC 1.13.11.34 | ALOX5 |
|  | EC 1.14.14.1 | CYP3A7; CYP3A7-CYP3A51P; CYP2F1; CYP4B1; CYP1B1; CYP2A7; CYP4X1; CYP4F11; CYP1A1; CYP1A2; CYP2C18; CYP4Z1; CYP2S1; CYP2A13; CYP3A43; CYP2J2; CYP2C8; CYP2D6; CYP3A5; CYP4F8; CYP4F12 |
|  | EC 1.14.15.3 | CYP4A22; CYP4A11 |
|  | EC 1.14.99.1 | PTGS2; PTGS1 |
|  | EC 3.1.1.4 | PLA2G1B; PLA2G6; PLA2G12B; PLA2G4F; PLA2G2E; PLA2G4E; PLA2G4D; PLA2G3; PLA2G2D; PLA2G2A; PLA2G4A; PLA2G12A; JMJD7-PLA2G4B; PLA2G4B; PLA2G2F; PLA2G10; PLA2G16; PLA2G4C; PLB1; PLA2G5 |
|  | EC 3.1.2.2 | BAAT; ACOT1; ACOT2; ACOT7; ACOT4 |
|  | EC 3.5.1.99 | FAAH; FAAH2 |
| PC(0:0/18:0) | EC 2.3.1.23 | LPCAT4; LPCAT3; LPCAT2; LPCAT1 |
| PC(18:0/0:0) | EC 2.3.1.43 | LCAT |
| PC(20:0/0:0) | EC 3.1.1.4 | PLA2G1B; PLA2G6; PLA2G12B; PLA2G4F; PLA2G2E; PLA2G4E; PLA2G4D; PLA2G3; PLA2G2D; PLA2G2A; PLA2G4A; PLA2G12A; JMJD7-PLA2G4B; PLA2G4B; PLA2G2F; PLA2G10; PLA2G16; PLA2G4C; PLB1; PLA2G5 |
| PC(17:0/0:0) | EC 3.1.1.5 | PLB1; PNPLA7; LYPLA1; ASPG; PNPLA6; PLA2G15; CLC; LYPLA2 |
| Xanthosine | EC 2.4.2.1 | PNP |
|  | EC 2.4.2.- | LARGE2; SIRT4; LARGE1 |
|  | EC 3.1.3.5 | NT5C3A; NT5E; NT5C2; ACPP; NT5C3B; NT5C1B; NT5C1B-RDH14; NT5C1A |
| cAMP | EC 3.1.4.17 | PDE10A; PDE1B; PDE1C; PDE3B; PDE1A; PDE2A; PDE3A |
|  | EC 3.1.4.53 | PDE4D; PDE8A; PDE4A; PDE4B; PDE4C; PDE7A; PDE8B; PDE11A; PDE7B |
|  | EC 4.6.1.1 | ADCY9; ADCY4; ADCY10; ADCY3; ADCY5; ADCY1; ADCY2; ADCY7; ADCY6; ADCY8 |
| SM(d18:1/22:0) | EC 2.3.1.24 | CERS5; CERS3 |
| SM(d18:1/23:0) | EC 2.7.8.27 | SGMS2; SGMS1 |
|  | EC 3.1.4.12 | SMPD2; SMPD4; SMPD1; ENPP7; SMPD3 |
| DHEAS | EC 2.8.2.2 | SULT2B1; SULT1C3 |
| androsterone sulfate + etiocholanolone sulfate | EC 3.1.6.2 | STS |
