# Supplementary figures and images for: Metabolic alterations in urine extracellular vesicles are associated to prostate cancer pathogenesis and progression
Source: J Extracell Vesicles. 2018 May 7;7(1):1470442. doi: 10.1080/20013078.2018.1470442 (PMC5944373; doi:10.1080/20013078.2018.1470442)

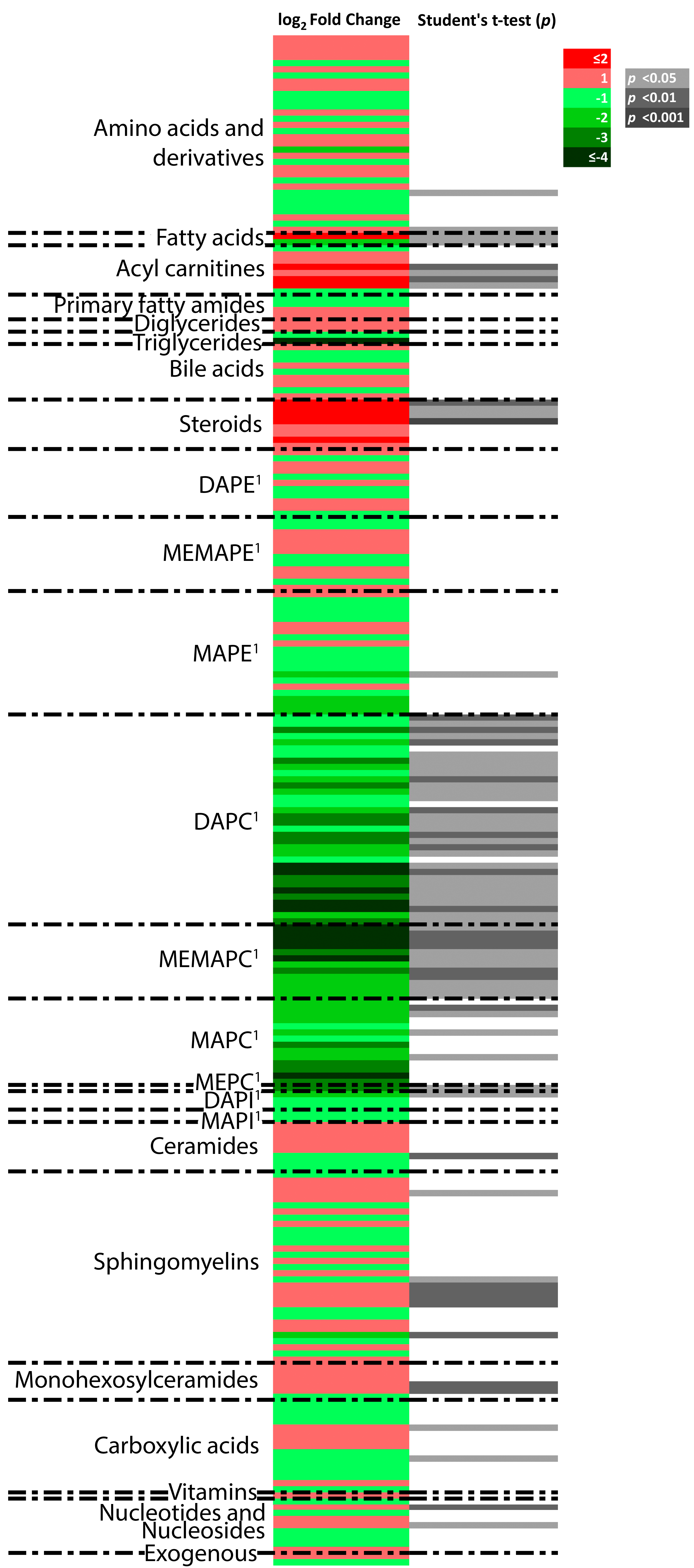

Supplement: Supplemental_files.zip [file ZJEV_A_1470442_SM6791.zip › Supplemental files/SUPPLEMENTAL_FIGURE_2.tif]

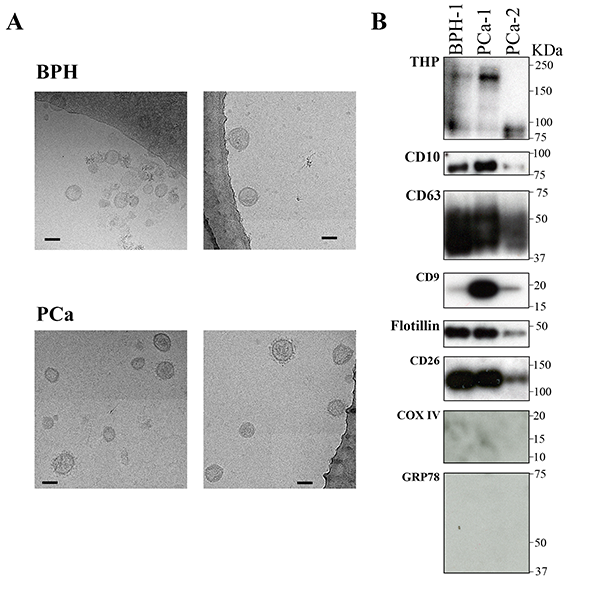

Supplement: Supplemental_files.zip [file ZJEV_A_1470442_SM6791.zip › Supplemental files/Supplementary_Figure 1.tif]
